# Multi robot exploration using an advanced multi-objective salp swarm algorithm for efficient coverage and performance

**DOI:** 10.1038/s41598-025-08194-w

**Published:** 2025-07-19

**Authors:** Ali El Romeh, Seyedali Mirjalili

**Affiliations:** 1 Centre for Artificial Intelligence Research and Optimization, Torrens University Australia, Melbourne, 3000 Australia; 2 Faculty of Electrical Engineering and Computer Science, VSB-Technical University of Ostrava, Ostrava, Czech Republic; 3 University Research and Innovation Center, Obuda University, Budapest, 1034 Hungary

**Keywords:** Multi-robot exploration, Salp swarm algorithm, Multi-objective optimization, Artificial Intelligence, Computational efficiency, Area coverage, Autonomous robotic systems, Electrical and electronic engineering, Mathematics and computing

## Abstract

This work introduces the Advanced Multi-Objective Salp Swarm Algorithm Exploration Technique (AMET), which is a novel optimization framework designed to enhance the efficiency and robustness of multi-robot exploration. AMET combines the deterministic structure of Coordinated Multi-Robot Exploration (CME) with the adaptive search capabilities of the Multi-Objective Salp Swarm Algorithm (MSSA) to achieve a balanced trade-off between exploration efficiency and mapping accuracy. To validate its effectiveness, AMET is compared to both multi-objective and single-objective exploration strategies, including CME combined with Multi-Objective Grey Wolf Optimizer (CME-MGWO), Multi-Objective Ant Colony Optimization (CME-MACO), Multi-Objective Dragonfly Algorithm (CME-MODA), and the single-objective CME with traditional Salp Swarm Algorithm (CME-SSA). The evaluation focuses on four critical performance metrics: runtime efficiency, exploration area coverage, mission completion resilience, and the reduction of redundant exploration. Experimental results across multiple case studies demonstrate that AMET consistently outperforms both single-objective and multi-objective counterparts, achieving superior area coverage, reduced computational overhead, and enhanced exploration coordination. These findings highlight the potential of AMET as a scalable and efficient approach for robotic exploration, providing a foundation for future advancements in multi-robot systems. The proposed method opens new possibilities for applications in search-and-rescue operations, planetary surface exploration, and large-scale environmental monitoring.

## Introduction

Robot exploration is a fundamental challenge in autonomous systems, involving the systematic traversal of unknown or partially known environments to construct accurate maps, identify critical regions, and optimize navigation paths. The significance of this problem spans multiple domains, including search-and-rescue operations in disaster-impacted areas, planetary surface exploration, and large-scale surveillance of hazardous or inaccessible environments. In these scenarios, autonomous robots offer clear advantages over human operators by reducing operational risks and improving the speed and quality of data acquisition. Depending on the complexity of the mission, exploration tasks may be executed using either a single-robot or a multi-robot system.

Single-robot exploration strategies often suffer from inherent limitations such as prolonged mission duration, increased failure risk due to the absence of redundancy, and constrained scalability. In response, multi-robot exploration (MRE) systems have emerged as an effective alternative, enabling cooperative exploration strategies that improve task efficiency and robustness in dynamic environments^1–3^. Multi-robot systems use distributed intelligence, which allows individual robots to collaboratively map unknown terrains, share exploration responsibilities, and enhance task completion rates. These systems facilitate parallelized environmental coverage, which leads to the reduction of mission time while enhancing resilience against system failures.

In multi-robot systems, inter-robot coordination is a critical factor influencing overall performance. Effective cooperation requires real-time data exchange to prevent collision conflicts and minimize redundant area scans. This coordination is achieved through shared mapping frameworks and adaptive path-planning strategies, which enables each robot to refine its trajectory based on dynamic environmental feedback. Such mechanisms allow for faster exploration rates, improved accuracy in environmental reconstruction, and enhanced fault tolerance in scenarios where communication disruptions or hardware malfunctions may occur.

Despite the advantages of MRE, optimizing multi-robot coordination under uncertain and dynamic conditions remains a complex problem. The field has increasingly focused on developing optimization-driven approaches, particularly swarm intelligence methodologies, to enhance exploration efficiency and task allocation^4,5^. The effectiveness of MRE systems directly impacts real-world applications, including environmental monitoring, planetary navigation, and automated reconnaissance^6,8,9^. Consequently, improvements in robot exploration algorithms directly translate to more scalable and deployable robotic solutions. However, the fundamental challenge in MRE lies in formulating exploration strategies that maximize terrain coverage while minimizing redundant traversal and inefficient path selection, making it a complex multi-objective optimization problem^10,11^.

Multi-robot exploration, like other optimization problems, can be approached using traditional deterministic methods or modern metaheuristic-based algorithms. Traditional approaches, including linear programming, nonlinear programming, and graph-based search methods, provide deterministic solutions but often suffer from computational intractability, particularly in high-dimensional and dynamically changing environments. Furthermore, such methods are prone to local optima entrapment, requiring extensive re-planning when environmental conditions shift. In contrast, metaheuristic algorithms offer adaptive and scalable alternatives by leveraging stochastic search mechanisms typically inspired by biological and swarm intelligence behaviors. These algorithms facilitate efficient search space exploration, escape from local optima, and adaptive response to environmental variations, making them well-suited for dynamic and large-scale MRE problems.

Among the various metaheuristic approaches, the Salp Swarm Algorithm (SSA) has demonstrated promising results due to its computational simplicity and efficiency in navigating complex search spaces ^12^. However, standard SSA primarily operates as a single-objective optimizer, limiting its applicability in multi-objective MRE scenarios^15^. To address these limitations, this study introduces the Advanced Multi-Objective Salp Swarm Algorithm Exploration Technique (AMET), which can be considered as an adaptation of SSA that extends its capabilities into a multi-objective optimization framework specifically designed for multi-robot exploration. AMET is structured to enhance exploration efficiency, area coverage, and operational robustness within cooperative robotic systems^16,17^.

**Figure 1:**
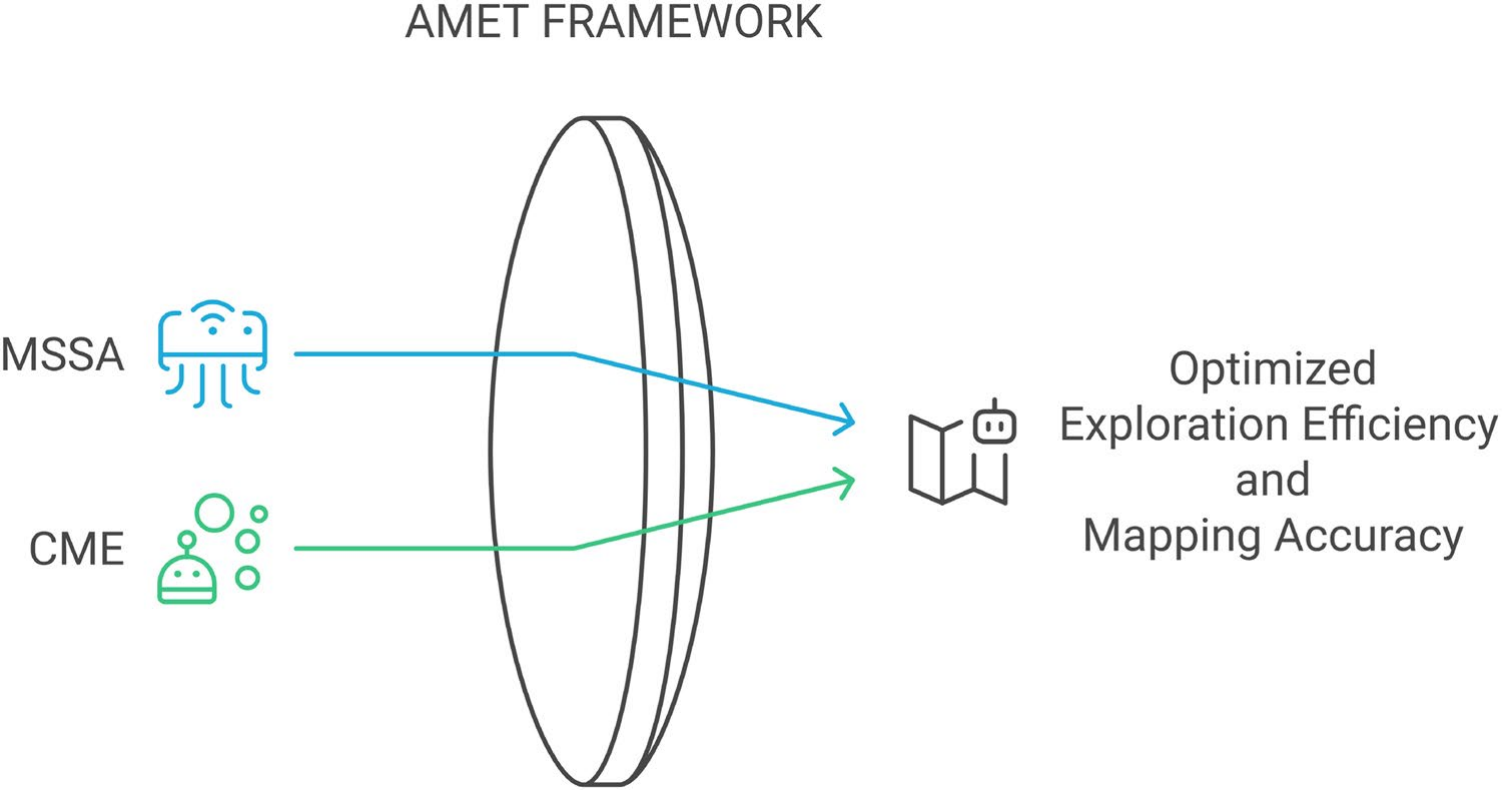
Conceptual diagram of the proposed AMET framework.

To provide a high-level understanding of the proposed AMET framework, a conceptual diagram is presented in Fig. 1. This diagram illustrates the integration of the Multi-Objective Salp Swarm Algorithm with Coordinated Multi-Robot Exploration to optimize exploration efficiency. The AMET framework enhances coordination, increases mapping accuracy, and reduces redundant traversal, with the ultimate goal of improving mission success in autonomous robotic applications. The optimization component enables adaptive search and decision-making, while the multi-robot coordination mechanism ensures efficient area coverage and task distribution.

The effectiveness of AMET is validated through a comparative analysis against established methodologies, including Coordinated Multi-Robot Exploration^18^, Multi-Objective Grey Wolf Optimizer^19^, Multi-Objective Ant Colony Optimizer^20^, and Multi-Objective Dragonfly Algorithm^21^. The evaluation focuses on computational runtime, percentage of explored area, resilience in mission completion, and the minimization of redundant area revisits^22,23^. The study contributes to the field by introducing AMET as a novel multi-objective optimization approach and providing an empirical evaluation of its performance against state-of-the-art exploration techniques^24,25^. The findings offer insights into the efficiency and adaptability of AMET in optimizing multi-robot exploration.

The proposed AMET introduces a novel hybrid framework that bridges the gap between deterministic coordination and adaptive optimization in multi-robot exploration. Unlike conventional metaheuristic-based MRE approaches that operate as black-box optimizers with limited environmental awareness, AMET tightly couples a deterministic task allocation model (CME) with the adaptive leader-follower dynamics of the MSSA algorithm. This combination allows AMET to dynamically balance exploration coverage and mapping accuracy by integrating context-aware decisions based on real-time cost maps and utility updates. Furthermore, we develop a real-valued solution encoding scheme tailored for 3D occupancy grids, which enables each robot to evaluate trade-offs between path cost and information gain at every decision point. To validate AMET’s effectiveness, we benchmark it against four state-of-the-art algorithms (CME-MGWO, CME-SSA, CME-MODA, and CME-MACO) across multiple simple and complex environments. Statistical analysis via the Wilcoxon rank-sum test confirms that AMET consistently achieves superior results. In addition, we provide a discussion on AMET’s practical applicability to real-world scenarios, such as disaster response and planetary mapping, where both reliability and scalability are essential. To our knowledge, this is the first work to explicitly integrate deterministic CME planning with adaptive multi-objective optimization in a unified exploratory framework.

The rest of the paper is organized as follows. Section 2 provides a comprehensive overview of the current research landscape in multi-robot exploration, which highlights recent advances and identifys the gaps addressed in this study. Section 3 discusses the theoretical underpinnings of the approach and details the development and structure of AMET. Section 4 presents the results and analysis of empirical tests. Finally, Section 5 summarizes the findings, discusses limitations, and suggests directions for future research.

## Literature review

Recent advancements in MRE have drawn significant attention due to their wide-ranging applications across various industries. These developments have been particularly notable in human-robot collaborative systems, where autonomous robots assist in exploring unknown terrains that are otherwise inaccessible or hazardous for humans^28,29^. Such such robotic systems, tasks such as planetary surface navigation, large-scale environmental monitoring, and search-and-rescue operations can be conducted with greater efficiency and safety^27^. Multi-robot systems, often supervised by human operators, enhance these capabilities by covering larger areas more effectively and reducing the dependency on human intervention^26^. As a result, research in this domain has increasingly focused on optimizing the deployment and coordination of these robotic systems to achieve higher efficiency and adaptability.

One of the primary advantages of multi-robot systems over single-robot approaches is their ability to coordinate exploration in complex environments while mitigating the risks associated with system failures. Unlike a single robot that must individually navigate and map its environment, a multi-robot system can divide the task across multiple agents, which allows for simultaneous data collection and faster environmental coverage^31^. This parallelized approach significantly reduces mission duration and enhances the robustness of exploration tasks. However, ensuring effective coordination among multiple robots introduces several challenges, particularly in communication, collision avoidance, and redundant scanning.

To manage multi-robot systems, two primary control strategies have been investigated: centralized and decentralized control. Centralized approaches involve a single control unit that governs all robots in the system, allowing for precise coordination but suffering from scalability limitations and vulnerability to a single point of failure. In contrast, decentralized control (especially in swarm robotics) enables autonomous decision-making at the individual robot level, which leads to greater flexibility and fault tolerance. Swarm robotic systems, inspired by biological collective behaviors, rely on local interactions between robots and their environment, which makes them highly adaptable to dynamic conditions. Although programming swarm behavior presents additional complexities, leveraging collective intelligence and distributed processing reduces the computational burden on individual robots, improving overall system efficiency.

When optimizing robotic exploration, algorithms can be broadly classified into deterministic and stochastic approaches. Deterministic algorithms rely on predefined rules and fixed decision-making criteria, ensuring that the same input will always produce the same outcome. While these methods offer predictability and computational efficiency, they often struggle with adaptability in high-dimensional or dynamically changing environments, where they can become trapped in local optima. On the other hand, stochastic algorithms, particularly meta-heuristic methods, incorporate randomness into their search process, which allows for greater exploration diversity and a higher probability of escaping local optima. Meta-heuristic techniques, inspired by biological and evolutionary processes, provide more robust solutions for large-scale and complex environments but at the cost of higher computational overhead and variable convergence rates. Despite these trade-offs, their ability to dynamically adjust exploration strategies makes them particularly well-suited for multi-robot systems operating in unknown and dynamic terrains.

Early studies in multi-robot exploration established the foundational principles of task allocation, coordination, and environmental coverage, forming the basis for modern optimization techniques^32^. As research evolved, the integration of heuristic and meta-heuristic approaches significantly improved the adaptability and efficiency of MRE methodologies, which extended their applications to real-world challenges such as environmental surveillance, industrial automation, and autonomous reconnaissance. These advancements highlight the growing importance of flexible, scalable, and intelligent algorithms in addressing the limitations of traditional rule-based approaches.

The subsequent sections examine the three main categories of multi-robot exploration algorithms: deterministic, meta-heuristic, and hybrid approaches. Given the strong emphasis on meta-heuristic optimization techniques in this study, a dedicated subsection will focus on their role in improving exploration performance and adaptability.

### Coordinated multi-robot exploration deterministic methods

In robotics research, there has been a significant shift toward multi-robot exploration, which highlights its growing importance in practical applications. As environments become more complex, effective coordination among multiple robots is essential to ensure efficient exploration. Deterministic methods have traditionally played a fundamental role in addressing this challenge by providing rule-based decision-making frameworks that optimize the allocation of exploration tasks. These methods aim to balance the trade-off between computational efficiency and exploration effectiveness, which ensurs that robots systematically cover unknown environments while minimizing redundant movements.

A key advantage of deterministic approaches is their ability to partition an environment into predefined sections, allowing robots to follow structured navigation plans. This methodology ensures predictability and repeatability, which makes it particularly useful in scenarios where real-time adaptability is less critical. One of the earliest and most widely used deterministic algorithms is the coverage algorithm proposed by Galceran and Carreras^34^. Their method divides the exploration space into discrete cells, assigning each robot a specific region to explore. Once a robot completes its designated section, it transitions to adjacent unexplored cells, ensuring systematic coverage. While this method is effective in structured environments with minimal obstacles, its efficiency decreases significantly in dynamic or large-scale exploration tasks, where real-time path adjustments are necessary.

To improve deterministic exploration strategies, Wang and Syrmos^35^ introduced the sweep algorithm, which organizes robots into zone-based formations, allowing them to traverse the environment in a predefined sweeping pattern. This approach is particularly well-suited for low-obstacle environments, where obstacles do not obstruct movement or require adaptive rerouting. However, in more complex terrains, where obstacles introduce unpredictable constraints, sweep-based approaches become inefficient as they lack the capability to dynamically adjust their routes. Further advancements in deterministic exploration introduced distributed mapping techniques, which allow multiple robots to operate using shared environmental data. Andries and Charpillet^32^ proposed a taboo-list strategy, which integrates global exploration maps, variable sensor vision, and optimized task distribution to prevent robots from revisiting previously explored areas. By incorporating real-time coordination strategies, their approach mitigates redundant scanning and premature convergence, which are common limitations in classical deterministic methods.

Despite their structured nature, deterministic algorithms struggle with scalability and adaptability, particularly in unpredictable or dynamically changing environments. Their reliance on predefined rules makes them prone to local optima entrapment, where robots repeatedly follow the same patterns without discovering more efficient paths. Altering the environment such as modifying the map structure is not always a feasible solution, making these methods less suitable for large-scale or evolving exploration tasks.

These limitations have prompted recent research to focus on stochastic approaches, particularly metaheuristic algorithms, which introduce adaptive search strategies and probabilistic decision-making to overcome the rigidity of deterministic models. The next section explores metaheuristic optimization techniques, which provide greater flexibility and robustness in multi-robot exploration by integrating randomized search mechanisms that enhance overall exploration efficiency.

### Metaheuristic algorithms

Metaheuristic algorithms have gained widespread attention in various fields due to their ability to perform global search, escape local optima, and optimize complex, high-dimensional problems without requiring gradients. Unlike deterministic approaches, which rely on predefined rules, metaheuristics benefit from probabilistic and evolutionary strategies to explore and exploit the search space efficiently. These methods have proven particularly effective in multi-robot exploration, where dynamic environments and uncertainty demand adaptive decision-making and flexible navigation strategies. Several well-regarded metaheuristic algorithms have been widely used in optimization problems, including Genetic Algorithm (GA), Particle Swarm Optimization (PSO), and Grey Wolf Optimizer (GWO). These methods have demonstrated success in solving a variety of optimization challenges, ranging from industrial process optimization to robotic path planning.

The Genetic Algorithm (GA), inspired by the principles of natural selection and genetic evolution, iteratively refines a population of candidate solutions through selection, crossover, and mutation operations^33^. This process allows GA to continuously improve solutions over multiple generations, making it effective in exploring large, complex search spaces. The Particle Swarm Optimization (PSO) algorithm^30^, on the other hand, is based on the collective behavior of bird flocking and fish schooling. By iteratively updating the positions of candidate solutions based on velocity and personal-best memory, PSO efficiently navigates nonlinear and high-dimensional search landscapes^37^. The Grey Wolf Optimizer (GWO), developed by Mirjalili et al.^36^, simulates the leadership hierarchy and hunting behavior of grey wolves to balance exploration and exploitation within the search space. The multi-objective variant (MOGWO) extends this principle to simultaneously optimize multiple conflicting objectives, making it suitable for multi-robot coordination problems, where trade-offs between coverage efficiency and energy consumption must be considered^19^.

A more recent and increasingly popular metaheuristic method is the Salp Swarm Algorithm (SSA)^12^. Inspired by the movement patterns of oceanic salps, SSA offers a fast-converging optimization strategy that has shown strong performance in single and multi-objective search problems. To enhance its adaptability, an advanced multi-objective variant, MSSA, was later introduced to preserve solution diversity and maintain Pareto-optimal fronts^12^. Through leader selection mechanisms and archive maintenance, MSSA effectively handles multi-objective optimization challenges while ensuring robust exploration.

Metaheuristic algorithms have demonstrated strong potential in multi-robot exploration, particularly in scenarios requiring dynamic path planning, real-time adaptation, and efficient coverage of unknown terrains. Their ability to efficiently navigate search spaces, avoid local optima, and converge toward optimal solutions makes them a versatile and scalable choice for robotic applications, ranging from continuous function optimization to discrete and mixed-integer problems.

In addition to swarm-based metaheuristics, adopted evolutionary algorithms such as the Non-dominated Sorting Genetic Algorithm II (NSGA-II)^13^ and the Multi-Objective Evolutionary Algorithm based on Decomposition (MOEA/D)^14^ have been extensively applied to multi-objective optimization problems. NSGA-II is known for its fast non-dominated sorting and crowding distance mechanisms, which ensure diversity and convergence, while MOEA/D decomposes the problem into scalar optimization subproblems to facilitate parallel optimization. Although effective in many applications, these algorithms often require extensive parameter tuning and computational overhead, particularly in high-dimensional or real-time robotic contexts. By contrast, the AMET framework integrates a lightweight stochastic optimizer (MSSA) with a deterministic exploration backbone (CME), which offers a more scalable and adaptive solution for multi-robot exploration in dynamic environments.

As the field progresses, the distinction between single-objective and multi-objective optimization strategies has become increasingly relevant in multi-agent robotic systems. The next section examines the key differences between these two approaches and their implications for coordinated multi-robot exploration.

### Contrasting multi-agent single objective and multi-agent multi-objective algorithms

In multi-agent optimization, the distinction between single-objective and multi-objective algorithms is fundamental to understanding how different approaches impact decision-making, convergence behavior, and solution diversity^7^. Single-objective algorithms are designed to optimize a single criterion, ensuring that the algorithm seeks one global optimal solution. Since only one objective is considered, comparing solutions is straightforward using relational operators, making evaluation and selection computationally efficient. These algorithms are well-suited for problems where a clear, singular goal, such as minimizing travel distance or maximizing energy efficiency, is the primary focus.

Multi-objective optimization, on the other hand, introduces a higher level of complexity, as it requires the simultaneous optimization of multiple, often conflicting objectives. Instead of converging to a single best solution, multi-objective algorithms produce a set of optimal trade-off solutions, known as Pareto-optimal solutions. Each solution within this set represents a unique balance between competing objectives, which ensures that no further improvements can be made in one objective without negatively affecting another. Unlike single-objective algorithms, where solutions can be ranked in absolute terms, multi-objective optimization demands more sophisticated evaluation techniques, such as Pareto dominance, hypervolume indicators, and diversity preservation mechanisms.

The fundamental difference between these two classes of algorithms lies in their solution representation, convergence criteria, and application domains. Single-objective algorithms perform well in tasks where a singular metric defines success, making them computationally efficient and easier to implement. In contrast, multi-objective algorithms are indispensable in real-world scenarios where multiple factors (such as energy efficiency, path length, coverage efficiency, and collision avoidance) must be optimized simultaneously. These algorithms provide a holistic approach to decision-making, ensuring that multiple system requirements are considered in an optimal manner.

Given the growing complexity of robotic exploration tasks, the transition from single-objective to multi-objective frameworks has been increasingly favored in recent research. The ability to handle multiple trade-offs dynamically allows multi-objective algorithms to better model real-world constraints, making them particularly valuable for coordinated multi-robot exploration. The next section explores hybrid approaches, which integrate deterministic and metaheuristic methods to further enhance exploration performance.

### Coordinated multi-robot exploration hybrid methods

Efficient exploration and mapping of unknown and cluttered environments are critical for the effective deployment of multi-robot systems in various real-world applications. Traditionally, research has focused on deterministic and metaheuristic algorithms separately, with deterministic methods offering structured task allocation and systematic coverage and metaheuristic techniques introducing adaptive exploration capabilities. However, these two approaches have inherent limitations. Deterministic methods often struggle with scalability and adaptability, while metaheuristics may suffer from slow convergence and computational overhead. To address these challenges, researchers have explored hybrid optimization frameworks, integrating the strengths of both deterministic and stochastic methods to enhance the performance of multi-robot exploration.

One of the earliest hybrid approaches was introduced by Albina and Lee^38^, who combined the CME and GWO algorithms to optimize robot trajectories in exploration and mapping tasks. Their results demonstrated that the hybrid method achieved complete coverage of the environment more efficiently compared to purely deterministic strategies. Similarly, Gul et al.^39^ developed a hybrid framework that integrated CME with the Frequency Modified Hybrid Whale Optimization Algorithm (FMH-WOA), which further enhanced coverage performance and computational efficiency. Both studies confirmed that hybrid approaches consistently outperform conventional deterministic methods by improving adaptability and optimizing robot movement in real-time.

To extend hybrid strategies to more challenging scenarios, Gul et al.^40^ introduced the Coordinated Multi-Robot Exploration Aquila Optimizer (CME-AO), designed specifically for barrier-filled environments. Their results showed that the hybrid approach outperformed both traditional CME strategies and standalone metaheuristic techniques in complex, constrained exploration spaces. In another work, Gul et al.^41^ presented the Hybrid Stochastic Optimizer (HSO), which combined deterministic CME with the Arithmetic Optimization Algorithm (AOA) to enhance coverage efficiency and reduce search time. These studies highlighted the growing importance of integrating deterministic and stochastic optimization to improve multi-robot exploration in diverse environments.

Building upon these hybrid methodologies, our previous research^25^ explored the integration of CME with the SSA algorithm, leading to the CME-SSA hybrid approach. Experimental results confirmed that this integration significantly improved exploration efficiency, search adaptability, and convergence speed compared to standalone algorithms. Further extending this work, we introduced the Hybrid Vulture-Coordinated Multi-Robot Exploration (HVCME) algorithm in^43^, where the African Vulture Optimization Algorithm (AVOA) was combined with CME to optimize exploration strategies. This hybrid model demonstrated superior coverage, time efficiency, and robustness, confirming that hybrid optimization significantly improves exploration performance in dynamic environments. However, in both CME-SSA and HVCME, the focus was limited to single-objective optimization, which left room for further advancements in multi-objective hybrid exploration strategies.

Despite these advancements, most hybrid approaches in multi-robot exploration have primarily focused on single-objective optimization, limiting their applicability in real-world scenarios that require balancing multiple conflicting objectives. There is a noticeable gap in the literature when it comes to scalable and adaptable multi-objective hybrid exploration algorithms, as highlighted in^25,38,39^. To bridge this gap, this study introduces the Advanced Multi-Objective Salp Swarm Algorithm Exploration Technique (AMET), which extends previous research by incorporating multi-objective optimization principles into hybrid frameworks^43^. The introduction of AMET represents a significant step toward improving scalability, adaptability, and efficiency in multi-robot exploration, ensuring optimized decision-making across multiple exploration objectives.

The next section presents the problem formulation and the proposed AMET framework, detailing its theoretical foundation and algorithmic implementation.

## Problem formulation and proposed method

This section first discusses the process of defining multi-objective multi-robot exploration problem followed by solution encoding. Then, the CME and MSSA algorithms are presented in details. Finally, the section provides the details of the proposed AMET framework.

### Problem definition and solution encoding

In this study, the multi-robot exploration problem is formulated as an optimization problem, where the primary objective is to maximize exploration efficiency and environmental coverage while ensuring an optimal navigation strategy. The task requires robots to systematically explore an unknown environment, construct an accurate map, and return to a designated starting position with the mapped information. To achieve this, the environment is discretized into an occupancy grid, where each cell represents a specific region of the environment with associated cost and utility values that influence the robots’ movement decisions.

The environment is modeled as a three-dimensional (3D) occupancy grid, where cells are defined by coordinate points (x, y, z). Each cell is assigned a probability value, $Prob_occ_{xyz}$, representing the likelihood of the cell being occupied by an obstacle. This probability serves as a key factor in path planning and collision avoidance, guiding the robots toward unexplored or less obstructed areas while minimizing redundant movements. Given the constraints imposed by unknown terrains, the robots must adapt their exploration strategies in real time, dynamically adjusting their trajectories based on sensor inputs and previously explored regions.

The solution to this optimization problem is represented as a sequence of robot positions and movements within the occupancy grid. At each iteration, robots evaluate neighboring cells based on cost and utility metrics, selecting the most optimal path for exploration. This decision-making process is influenced by factors such as exploration potential, obstacle density, and inter-robot communication to ensure efficient spatial distribution of the robots. The following key components are used in the solution encoding:

- Occupancy Grid Map: A grid where each cell has a probability of being occupied, costs for traversal, and utility values for exploration.
- Robot Position Updates: Robots move based on a deterministic cost function that considers the probability of occupancy, the Euclidean distance between cells, and the utility values of the cells.
- Utility Value Assessment: The utility of each cell is updated as robots explore the environment, decreasing as cells are visited and increasing for frontier cells that represent new, unexplored areas.
- Multi-Objective Optimization: The proposed AMET balances the trade-offs between exploration efficiency and mapping accuracy by optimizing these objectives simultaneously.

The above problem formulation and solution encoding allow the real-valued optimizer to effectively navigate the robots through the environment. To ensure maximum area coverage and efficient exploration, this process involves continuous updates to the robots’ positions based on real-time evaluations of costs and utilities, which allows for adaptive and dynamic exploration strategies.

### Deterministic CME

A fundamental aspect of AMET is the deployment of multiple autonomous robots to efficiently map an unknown environment. Exploration strategies in multi-robot systems are typically classified into centralized and decentralized approaches. In centralized exploration, all robots operate using a shared global map, enabling real-time updates on exploration progress. This approach fosters inter-robot communication, which allows robots to coordinate their movements and avoid redundant scanning. By contrast, decentralized exploration relies on each robot constructing its own local map, with information exchanged only when robots cross paths. While this decentralized approach reduces the complexity of coordination, it may lead to inefficient coverage and redundant exploration, as individual robots lack access to the collective mapping data of the team.

This study employs the centralized exploration strategy as part of the AMET framework, leveraging its advantages in coordination and data sharing to improve exploration efficiency. Within this framework, AMET dynamically updates utility values and travel costs for each robot, ensuring adaptive path selection and optimized exploration patterns. The environment is modeled using an occupancy grid representation, where each cell contains numerical values indicating obstacle probability, utility score, and travel cost. Robots, initially placed in an unknown indoor setting with limited-range sensors, systematically identify frontier cells, which serve as reference points for expanding the explored map. Given the constraints imposed by sensor limitations, robots can only perceive and update cells within their immediate sensing range, requiring an iterative update mechanism to progressively construct a complete map of the environment.

A crucial component of AMET is the definition and formulation of the cost function, which governs path selection and exploration priority. The effectiveness of multi-robot exploration depends on minimizing traversal costs while maximizing coverage efficiency. In the AMET approach, determining the most optimal path from the robot’s current position to all identified frontier cells is essential for efficient exploration. This is achieved through a deterministic cost function that integrates occupancy grid probability, sensor range, and Euclidean distance between waypoints. The initial cost function, as defined in Eq. (1), accounts for grid occupancy probability, sensor constraints, and distance metrics. To ensure path efficiency, previously explored cells contribute historical cost values to influence current navigation decisions. However, for frontier cells, no backward cost accumulation is applied, as frontier regions serve as expansion points rather than revisited areas, as outlined in Eq. (3).

The three-dimensional occupancy grid map uses coordinates (*x*, *y*, *z*), where *x*, *y*, and *z* denote the cell’s position along the respective axes. Utility and cost values are primarily stored on the x-y plane, setting *z* to zero. This grid map depicts the environment the robot navigates. The terms $\Delta x$, $\Delta y$, and $\Delta z$ are used to denote the incremental changes or shifts in the cell’s position along each of these axes. These increments are essential for exploring neighboring cells in the grid. They represent a step in any direction (positive, negative, or zero) along the respective axis, allowing the algorithm to evaluate and update the cost function by considering all neighboring cells around a given position in the grid.

The cost of traversing a cell (*x*, *y*, *z*) inversely relates to its occupancy probability, $\text {{Prob\_occ}}_{xyz}$. The algorithm performs a two-step process (Eqs. 1 and 2) to identify the least-cost path^18^.

1 $$\begin{aligned} C(x,y,z) = {\left\{ \begin{array}{ll} 0, & \text {if } (x,y,z) \text { is the robot's location},\\ \infty , & \text {otherwise} \end{array}\right. } \end{aligned}$$

The algorithm updates each grid cell’s status at (*x*, *y*, *z*) iteratively.

2 $$\begin{aligned} C(x,y,z) = \min \biggl [ C_{x+\delta x, y+\delta y, z+\delta z} + (\Delta x^2+\Delta y^2+\Delta z^2) \times \text {{Prob\_occ}}_{x+\Delta x, y+\Delta y, z+\Delta z} \biggr ] \end{aligned}$$

3 $$\begin{aligned} C(x,y,z) = \min \left[ (\Delta x^2+\Delta y^2+\Delta z^2) \times \text {{Prob\_occ}}_{x+\Delta x,y+\Delta y,z+\Delta z}\right] \end{aligned}$$

where $\delta x, \delta y, \delta z \in [-1, 0, 1]$, and $\text {{Prob\_occ}}_{x+\Delta x,y+\Delta y,z+\Delta z} \in [0, \text {{Max\_occ}}]$, with $\text {{Max\_occ}}$ representing the highest occupancy probability. Selecting the next position for a robot involves identifying the neighboring cell with the lowest cost, ensuring an optimal exploration trajectory. In single-robot systems, decision-making primarily focuses on minimizing traversal costs to improve localization. However, in multi-robot systems, effective exploration requires organized collective behavior, where multiple robots coordinate movements to maximize coverage while minimizing redundancy. The Coordinated Multi-Robot Exploration (CME) framework, integrated within AMET, facilitates efficient task distribution by ensuring that robots explore distinct regions rather than overlapping paths. This approach enhances exploration efficiency and spatial coverage, making it superior to single-robot strategies.

In addition to cost considerations, utility values play a crucial role in determining the exploration priority of each grid cell. At the initial stage of exploration, all cells are assigned equal utility values, reflecting their unknown status. As robots traverse the environment, these values are dynamically updated based on exploration progress, as defined in Eq. (4). Specifically, the utility values of frontier cells decrease as they are explored, incentivizing robots to prioritize unexplored areas with higher utility scores. This strategy guides robots toward new regions, ensuring a progressive expansion of environmental knowledge and an optimized exploration trajectory.

The cost of a grid cell is influenced by its proximity to the robot, meaning that cells closer to the robot are less costly to visit. However, the utility function introduces an additional decision layer, encouraging robots to explore regions with higher informational gain rather than simply following the shortest path. The utility of a frontier cell is determined by its immediate surroundings and the number of robots targeting it. By incorporating both cost-based decision-making and utility-driven exploration, AMET balances efficient coverage with strategic path selection, ensuring that multi-robot systems explore new areas systematically while minimizing redundant scanning. The resulting approach enhances overall mapping accuracy and exploration efficiency, as detailed in Eq. (4).

4 $$\begin{aligned} V_{xyz}^t = V_{xyz}^{t-1} - \sum _{x=1}^{n-1} \left| \text {Prob\_occ}_{xyz}^{\text {current}} - \text {Prob\_occ}_{xyz}^{\text {robot}} \right| \end{aligned}$$

Here, $V_{xyz}^t$ denotes the current utility value of a cell, reflecting its exploration significance. $V_{xyz}^{t-1}$ represents the utility of the same cell in the previous phase. $\text {Prob\_occ}$ indicates the occupancy probability of the cell at *x*, *y*, *z*. The sum captures the variation in perceived occupancy probability from the robot’s perspective at different times. To enhance exploration, the cell with the highest utility at iteration *t* is identified using Eq. (5), incorporating both the occupancy probability and the prior utility value of the cell.

5 $$\begin{aligned} \left( \text {cell}_{xyz}^*, t\right) = \text {argmax} \left\{ V_{xyz}^t - C_{xyz} \right\} \end{aligned}$$

For effective cooperative exploration, robots should start in close proximity, enabling sensor range overlap. This strategy allows them to spread out in various directions, targeting different locations, and thus lowering utility values. The test environments were confined to a 50m x 50m area with limited sensor range lengths. The explored regions are shown in blue, while obstacles are indicated in dark grey.

### Multi-objective salp swarm algorithm (MSSA)

The SSA algorithm^12^, introduced by Mirjalili et al., is a metaheuristic optimization method inspired by the swarming behavior of salps. In their natural environment, salps form chain-like structures in deep oceans to enhance foraging efficiency and coordinated movement. SSA models this behavior in a computational framework, distinguishing between leaders and followers, where the leader guides the exploration process while followers update their positions based on preceding salps. The MSSA algorithm extends this approach to multi-objective optimization, integrating Pareto-based selection mechanisms to handle competing objectives effectively. The algorithm maintains a repository of non-dominated solutions, similar to Multi-Objective Particle Swarm Optimization (MOPSO), which ensures a well-distributed set of Pareto-optimal solutions throughout the optimization process.

*Position Update for the Leading Salp:*

The position of the leading salp is updated using a modified equation, as shown in Eq. (6):

6 $$\begin{aligned} p_k^1 = {\left\{ \begin{array}{ll} T_k + d_1 ((U_k - L_k)d_2 + L_k) & \text {if } d_3 \ge 0.5, \\ T_k - d_1 ((U_k - L_k)d_2 + L_k) & \text {if } d_3 < 0.5, \end{array}\right. } \end{aligned}$$

where $p_k^1$ denotes the position of the leader and $T_k$ the target (food source) in the $k$-th dimension. $U_k$ and $L_k$ represent the upper and lower bounds in the $k$-th dimension. The terms $d_1$, $d_2$, and $d_3$ are random numbers, with $d_1$ defined in Eq. (7):

7 $$\begin{aligned} d_1 = 2e^{-\left( \frac{4m}{\text {MaxIter}}\right) ^2}, \quad d_2, d_3 \in [0, 1], \end{aligned}$$

where $m$ is the current iteration and MaxIter is the maximum number of iterations.

*Position Update for the Follower Salps:*

The followers’ positions are updated based on a modified version of Newton’s law of motion, shown in Eq. (8):

8 $$\begin{aligned} p_k^m = \frac{1}{2} b t^2 + v t, \end{aligned}$$

where $m$ is the current iteration ($m \ge 2$), $p_k^m$ is the position of the $m$-th follower in the $k$-th dimension, $v$ is the initial velocity, and $t$ represents time. The parameters $b$ and $v$ are determined as follows:

$$\begin{aligned} b = \frac{v_{\text {final}}}{v}, \quad v = \frac{p - p_0}{t}. \end{aligned}$$

Since $v = 0$ at initialization and considering iterations as time steps, the position update simplifies to the averaged form shown in Eq. (9):

9 $$\begin{aligned} p_k^m = \frac{p_k^m + p_k^{m-1}}{2}, \quad m \ge 2. \end{aligned}$$

This equation ensures smooth positional transitions, preventing abrupt changes that could lead to instability in the swarm. It also preserves chain formation, as each follower adapts its movement relative to its preceding salp. By maintaining this structured motion, the algorithm ensures that the swarm progresses in a cohesive manner, avoiding erratic jumps that could disrupt the search process.

To maintain a diverse set of non-dominated solutions, MSSA employs an adaptive repository that continuously updates throughout the optimization process. Each newly generated solution is evaluated against the current repository members based on Pareto dominance principles. If a salp outperforms an existing solution, the dominated solution is removed. When the repository reaches its maximum storage capacity, a density-based selection mechanism is triggered, prioritizing the removal of solutions from regions of high density within the Pareto front. This strategy ensures that the repository maintains an evenly distributed set of solutions, avoiding excessive clustering in any specific area of the objective space.

The repository also plays a crucial role in guiding salps toward unexplored regions of the search space. New candidate solutions are selected from sparsely populated areas of the Pareto front, thereby enhancing search diversity. This selection process follows a roulette wheel mechanism, which balances the trade-off between exploration and exploitation. By directing computational efforts toward underrepresented areas, the algorithm improves solution diversity and avoids premature convergence to suboptimal regions.

At the beginning of the optimization process, MSSA initializes a population of salps and assigns them positions within the predefined search space. These initial positions are constrained by the problem’s upper and lower bounds, ensuring that the search remains within feasible limits. Once initialization is complete, the algorithm evaluates the objective function values for each salp, identifying the non-dominated solutions within the population. These solutions are then stored in the repository, provided there is available space. If the repository is full, a maintenance procedure is initiated to regulate its contents.

During repository maintenance, solutions in crowded regions are identified and ranked based on their contribution to the diversity of the Pareto front. The algorithm employs a roulette wheel selection process to determine which solutions to remove, thereby making room for new non-dominated solutions. This dynamic regulation ensures that the repository remains diverse and well-distributed across the entire search space, preventing the algorithm from favoring specific regions disproportionately.

Once the repository is updated, the algorithm selects a food source from it, preferably from a less crowded region. This selection process is again guided by ranking and the roulette wheel method to encourage movement toward underexplored regions of the Pareto front. The leading salp and follower salps then update their positions according to the modified motion equations (Eqs. 6 and 9), ensuring that the swarm gradually moves toward the most promising solutions. If a salp exceeds the predefined search space boundaries during this update, it is repositioned back within feasible limits to maintain solution validity.

The MSSA algorithm iteratively executes these steps until a termination criterion is satisfied. This criterion may be a fixed number of iterations or a convergence threshold that signifies that further improvements in the solution set are negligible. The algorithm’s structured approach to repository maintenance, solution selection, and position updates ensures that it continuously refines the Pareto front while maintaining a robust balance between search space exploration and exploitation.

In summary, MSSA employs a specialized repository to preserve non-dominated solutions, preventing the loss of valuable solutions even when the population undergoes deterioration. Additionally, the algorithm actively directs its search toward less populated regions of the Pareto front, ensuring solution diversity. By inheriting key SSA characteristics, including its leader-follower structure, MSSA remains computationally efficient, with only a few essential parameters (such as $c_1$ and archive size) for simplicity. Despite its computational complexity of $O\bigl (t(d\cdot n + \text {Cof}\cdot n + M\cdot n^2)\bigr )$, MSSA has proven effective in generating well-distributed Pareto-optimal solutions in both benchmark and real-world scenarios. Its iterative procedure guarantees the continuous evaluation of fitness values, repository updates, and boundary constraint enforcement, ensuring a robust trade-off between exploration and exploitation in multi-objective optimization tasks.

**Figure 2:**
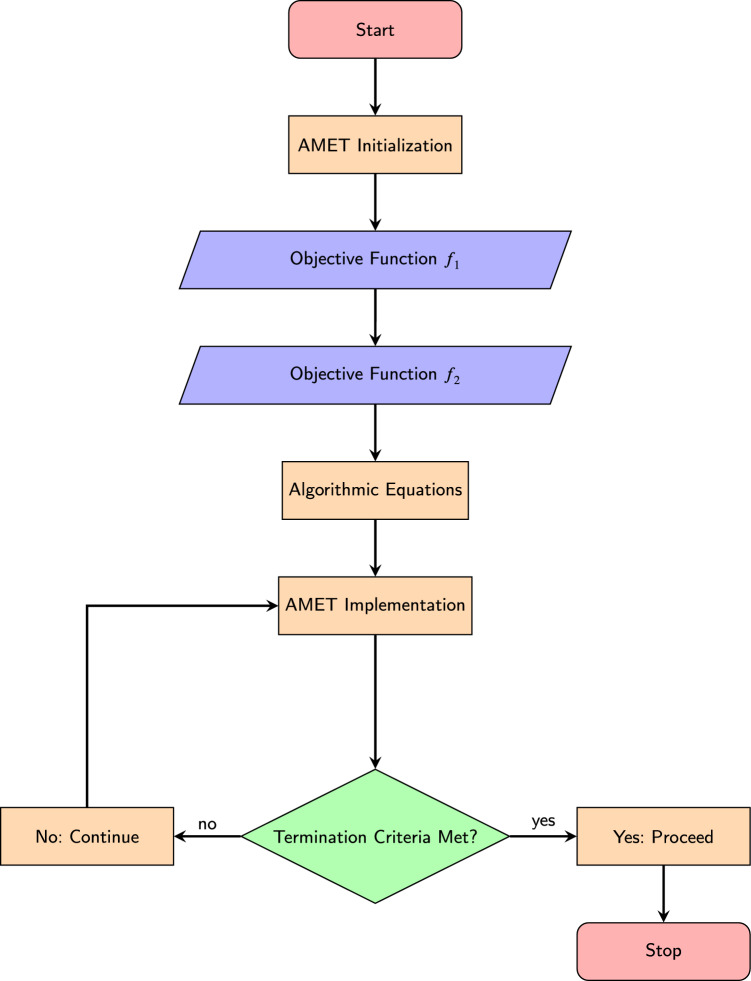
Flowchart of the AMET process.

### Proposed advanced multi-objective salp swarm algorithm exploration technique (AMET)

The proposed AMET integrates the structured task allocation mechanism of CME with the adaptability and optimization efficiency of MSSA. This hybrid approach is designed to enhance multi-robot exploration in a three-dimensional environment by ensuring a balance between exploration efficiency and mapping accuracy. Unlike traditional exploration strategies, which either focus on deterministic task allocation or stochastic search strategies in isolation, AMET leverages the strengths of both methodologies to maximize coverage and improve mapping precision.

AMET operates by simultaneously optimizing two key objective functions: maximizing the explored area and minimizing mapping uncertainty. The first objective function ensures that the robots explore as much of the environment as possible while also encouraging diversity in their trajectories. This is mathematically formulated in Eq. (10):

10 $$\begin{aligned} f_1 = \sum _{\text {all cells}} \text {ExploredStatus}(x,y,z) + \text {DiversityBonus}(x,y,z) \end{aligned}$$

Where ExploredStatus(*x*, *y*, *z*) is 1 if the cell at coordinates (*x*, *y*, *z*) has been explored, and 0 otherwise. The term DiversityBonus(*x*, *y*, *z*) introduces an additional incentive for robots to explore areas with higher environmental diversity, ensuring that exploration does not remain confined to predictable patterns or easily accessible locations. This encourages robots to move beyond immediate local optima, leading to a more evenly distributed exploration process. The *DiversityBonus*(*x*, *y*, *z*) term is computed using entropy-based spatial variance or local neighborhood entropy, encouraging exploration of high-variance regions. This prevents premature convergence and helps robots escape local optima.

The second objective function focuses on improving the precision and accuracy of the generated environmental map. This is achieved by minimizing uncertainty in the occupancy grid, ensuring that each mapped region provides a high level of detail and minimal ambiguity. The formulation for this objective is given in Eq. (11):

11 $$\begin{aligned} f_2 = -\sum _{\text {all cells}} \text {MapUncertainty}(x,y,z) \end{aligned}$$

Where MapUncertainty(*x*, *y*, *z*) quantifies the level of uncertainty or error associated with each explored cell. Lower uncertainty values correspond to higher map reliability, directly contributing to a more detailed and useful representation of the environment. By optimizing both exploration coverage and mapping accuracy, AMET effectively improves the overall utility of multi-robot exploration in complex and unknown terrains. The uncertainty is modeled based on the variance of occupancy probabilities across time. A lower variance implies better mapping confidence. The negative sign is used because we aim to minimize uncertainty.

The sequential process of AMET, from initialization to termination, is outlined in Fig. 2. This flowchart illustrates how the algorithm iterates through key phases, including objective function evaluation, adaptive optimization, and cost map updates, ensuring efficient multi-robot exploration.

The implementation of AMET involves an initialization step, where the cost map is defined to guide robots through the environment. The cost map assigns an initial value to each cell, representing the traversal difficulty and probability of encountering obstacles. This is initialized as shown in Eq. (12):

12 $$\begin{aligned} \text {CostMap}(x, y, z) = {\left\{ \begin{array}{ll} 0 & \text {if } (x, y, z) \text { is the robot's position}, \\ \infty & \text {otherwise}. \end{array}\right. } \end{aligned}$$

Once initialized, the cost update loop iteratively refines the map by updating the traversal cost based on the occupancy probability of neighboring cells. This ensures that the robots dynamically adapt their paths to avoid high-risk areas while prioritizing exploration in low-cost, high-information zones. The square root term reflects the Euclidean distance between the current and candidate cell. Multiplying by the occupancy probability penalizes riskier paths. This ensures safer and more efficient traversal. The cost update equation is defined in Eq. (13):

13 $$\begin{aligned} \text {CostMap}(x, y, z) = \min \Big \{ \text {CostMap}(x + \Delta x, y + \Delta y, z + \Delta z) + \sqrt{\Delta x^2 + \Delta y^2 + \Delta z^2} \times \text {ProbOcc}(x + \Delta x, y + \Delta y, z + \Delta z) \Big \} \end{aligned}$$

In addition to cost estimation, AMET integrates a dynamic utility update mechanism, ensuring that robots prioritize regions with higher exploratory significance. The utility function is formulated in Eq. (14):

14 $$\begin{aligned} \text {CellUtility}(x, y, z) = \text {PrevUtility} - \sum _{k=1}^{n-1} |\text {OccCurr}_{x,y,z} - \text {OccPrev}_{x,y,z}| \end{aligned}$$

This equation dynamically adjusts the utility value of each cell by considering changes in occupancy probability. As new regions are explored, the utility decreases for already visited locations while high-utility frontier cells are emphasized to drive further exploration. Also, this formula decreases utility as the occupancy of a cell becomes more certain (i.e., lower information gain). It favors frontier cells, which often have higher occupancy variance.

To determine the most optimal movement trajectory, AMET selects the next target cell using a decision rule that balances both utility and cost constraints, as shown in Eq. (15):

15 $$\begin{aligned} \text {TargetCell} = \mathop {\mathrm {arg\,max}}\limits \{ \text {CellUtility}(x, y, z) - \text {CostMap}(x, y, z) \} \end{aligned}$$

By maximizing the difference between cell utility and traversal cost, AMET ensures that robots prioritize unexplored regions while minimizing redundant movements.

Once the target selection is determined, AMET updates the leader robot’s position based on adaptive motion equations. The leader position update mechanism follows a probabilistic adjustment described in Eq. (16):

16 $$\begin{aligned} \text {LeaderPos}_j = {\left\{ \begin{array}{ll} \text {Target}_j + c_1 \times ((U_j - L_j) \times c_2 + L_j), & \text {if } c_3 \ge 0.5, \\ \text {Target}_j - c_1 \times ((U_j - L_j) \times c_2 + L_j), & \text {if } c_3 < 0.5. \end{array}\right. } \end{aligned}$$

To control the balance between exploration and exploitation, AMET adjusts parameters dynamically based on the iteration count, as defined in Eq. (17):

17 $$\begin{aligned} \text {Param}_1 = 2e^{-\left( \frac{4i}{\text {MaxIterations}}\right) ^2}, \quad \text {RandomParam}_2, \text {RandomParam}_3 \in [0, 1] \end{aligned}$$

For follower robots, AMET adopts a Newtonian motion model to update positions, given in Eqs. (18) and (19):

18 $$\begin{aligned} \text {FollowerPos}_j = \frac{1}{2} a t^2 + v_0 t, \quad \text {for } j \ge 2 \end{aligned}$$

19 $$\begin{aligned} \text {FollowerPos}_j = \frac{1}{2}(\text {FollowerPos}_j + \text {FollowerPos}_{j-1}) \end{aligned}$$

A further refinement is introduced by incorporating occupancy-based leader adjustments, ensuring that high-risk areas are weighted in decision-making, as shown in Eq. (20):

20 $$\begin{aligned} \text {LPos}_i = {\left\{ \begin{array}{ll} \text {ProbOcc}(x + \Delta x, y + \Delta y, z + \Delta z) + P_1 \times ((U_i - L_i) \times R_2 + L_i), & \text {if } R_3 \ge 0.5, \\ \text {ProbOcc}(x + \Delta x, y + \Delta y, z + \Delta z) - P_1 \times ((U_i - L_i) \times R_2 + L_i), & \text {if } R_3 < 0.5. \end{array}\right. } \end{aligned}$$

Robot movement decisions within AMET are based on decision variables derived from cost and utility mappings, summarized in Eq. (21):

21 $$\begin{aligned} \text {DecisionVar}_{x, y, z} = \text {Determined by CostMap and CellUtility} \end{aligned}$$

Finally, AMET is implemented with an initial setup phase, where roles and parameters are assigned to each robot based on their initial deployment region. The algorithm iteratively updates robot positions and exploration tasks, optimizing the trade-off between exploration efficiency $f_1$ and mapping accuracy $f_2$. The process continues until a predefined coverage goal is met or sufficient environmental data is collected.

Unlike traditional multi-objective algorithms such as MOEA/D and NSGA-II, which rely on decomposition-based or dominance-based sorting mechanisms, AMET incorporates an adaptive swarm intelligence model that directly embeds environmental structure through the deterministic CME layer. MOEA/D decomposes the problem into scalar subproblems and requires carefully tuned weight vectors, while NSGA-II employs non-dominated sorting and crowding distance to guide selection, which are both computationally expensive. In contrast, AMET leverages the lightweight and leader-follower dynamics of MSSA to ensure convergence and diversity, while the deterministic CME layer enables context-aware path planning and task allocation. This fusion results in faster convergence, lower redundant exploration, and reduced overhead, as CME pre-structures the search space for MSSA to optimize. Thus, the hybrid design enhances both exploration efficiency and computational tractability in complex robotic environments.

In the following section, several exploration scenarios are tested, comparing AMET’s efficiency and robustness against existing approaches. The results illustrate the effectiveness of the proposed methodology in improving exploration coverage, mapping accuracy, and computational efficiency across diverse environments.

## Results and discussion

This section presents the results of the AMET algorithm compared to other multi-objective heuristic approaches. The findings demonstrate that AMET consistently outperforms competing algorithms in terms of exploration efficiency, computational time, and reliability. To ensure a comprehensive evaluation of AMET’s performance, a series of test maps with varying complexities was employed. These maps were designed to simulate realistic exploration challenges, including obstacle-filled environments, open spaces, and irregular terrain structures. The comparative analysis was conducted against four existing algorithms, each integrating CME with a different meta-heuristic approach:

1. Coordinated Multi-Robot Exploration and Multi-Objective Grey Wolf Optimizer (CME-MGWO)
2. Coordinated Multi-Robot Exploration and Salp Swarm Algorithm (CME-SSA)
3. Coordinated Multi-Robot Exploration and Multi-Objective Dragonfly Algorithm (CME-MODA)
4. Coordinated Multi-Robot Exploration and multi-objective Ant Colony Optimization (CME-MACO)

These algorithms were evaluated based on key performance metrics, including execution time, proportion of explored area, and number of unsuccessful runs. All simulations were conducted on a standardized 50 m $\times$ 50 m map, with robots strategically positioned to ensure cooperative exploration. Color coding was used to distinguish explored areas, unexplored regions, and obstacles, providing a clear visual representation of robot movements.

Given the stochastic nature of meta-heuristic algorithms, the Wilcoxon rank-sum test was employed to statistically validate the significance of the observed differences in performance. This non-parametric test was chosen due to its effectiveness in comparing two independent algorithmic distributions, ensuring that differences in performance were not due to random chance.

To quantitatively assess exploration efficiency, the proportion of the explored area was calculated using the following formula:

$$\begin{aligned} E_A = \frac{V_{\text {initial}} - V_{\text {explored}}}{V_{\text {initial}}} \times 100 \end{aligned}$$

where $V_{\text {initial}}$ represents the total number of grid cells available for exploration before the simulation begins, and $V_{\text {explored}}$ represents the number of cells successfully explored by the robots. This metric provides a direct measure of exploration efficiency, allowing for a standardized comparison across different algorithms.

To ensure statistical reliability, the central limit theorem was considered^42^, leading to the selection of a sample size of 30, which is generally sufficient to approximate a normal distribution of results. Each algorithm was subjected to 500 iterations, with simulations repeated 30 times per algorithm to account for variance introduced by stochasticity. Given the inherent randomness of meta-heuristic approaches, each simulation produced slightly different outcomes, making repeated runs essential for accurate performance evaluation.

During the simulations, each robot was assigned a distinct color for easy identification, ensuring that the interactions between robots and their respective exploration trajectories could be clearly analyzed. Upon completing all experimental runs, the performance of AMET was compared against the four benchmark algorithms by analyzing the number of explored grid cells, total exploration time, and the frequency of incomplete runs.

The next section presents the experimental results obtained in both simple and complex environments and discusses their implications in terms of exploration efficiency, computational performance, and scalability.

**Figure 3:**
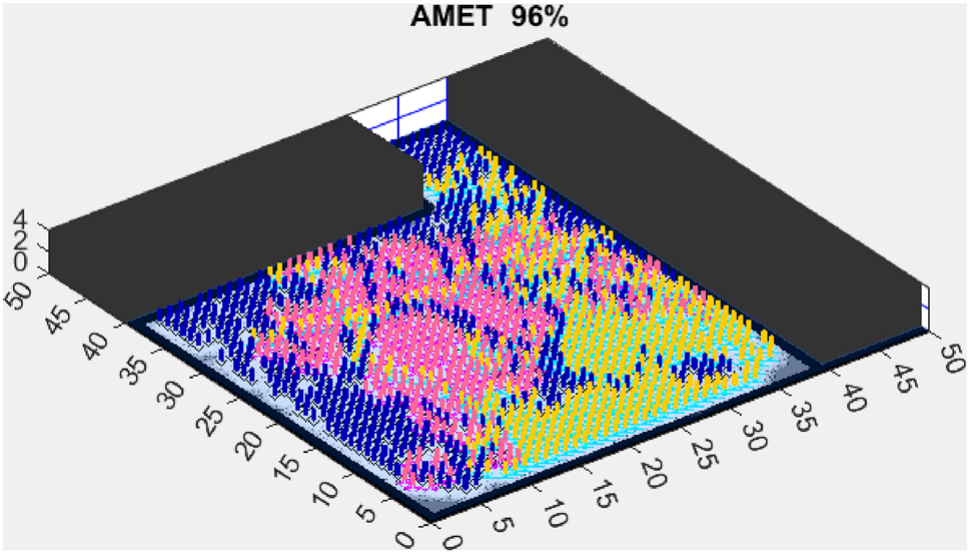
AMET 96% exploration rate.

**Figure 4:**
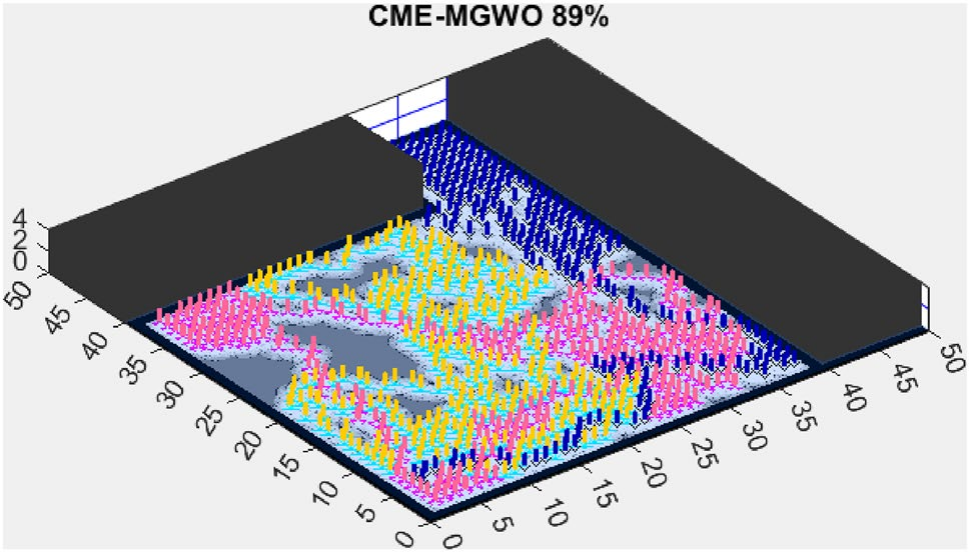
CME-MGWO 89%.

**Figure 5:**
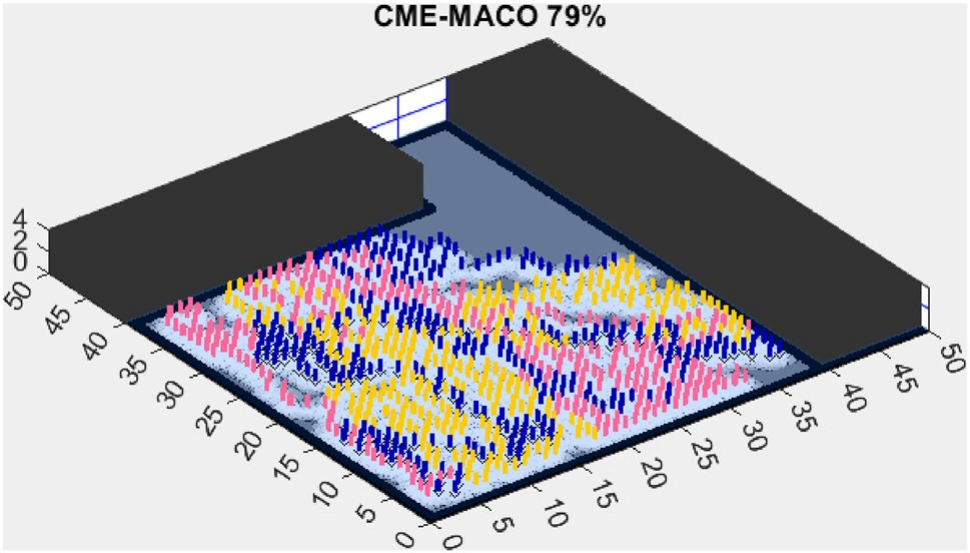
CME-MACO 79%.

**Figure 6:**
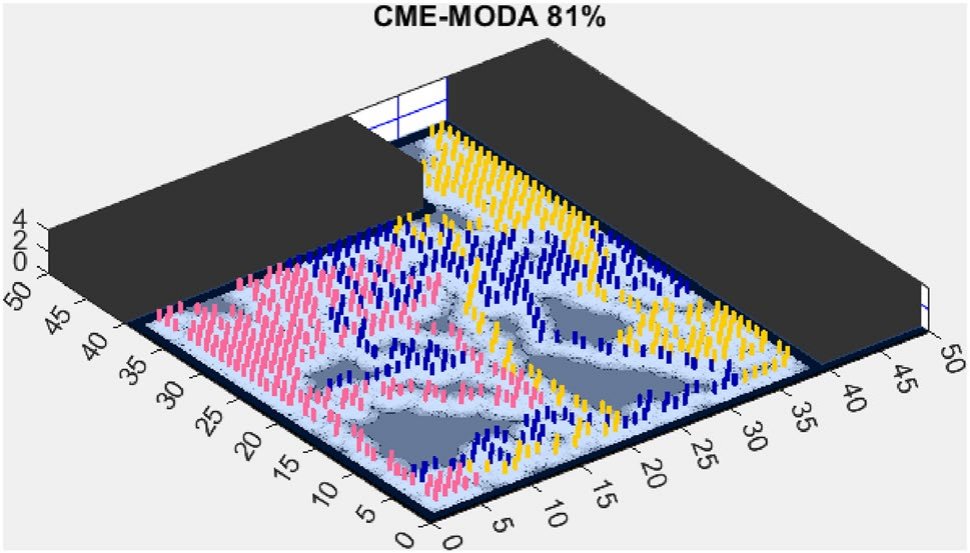
CME-MODA 81%.

**Figure 7:**
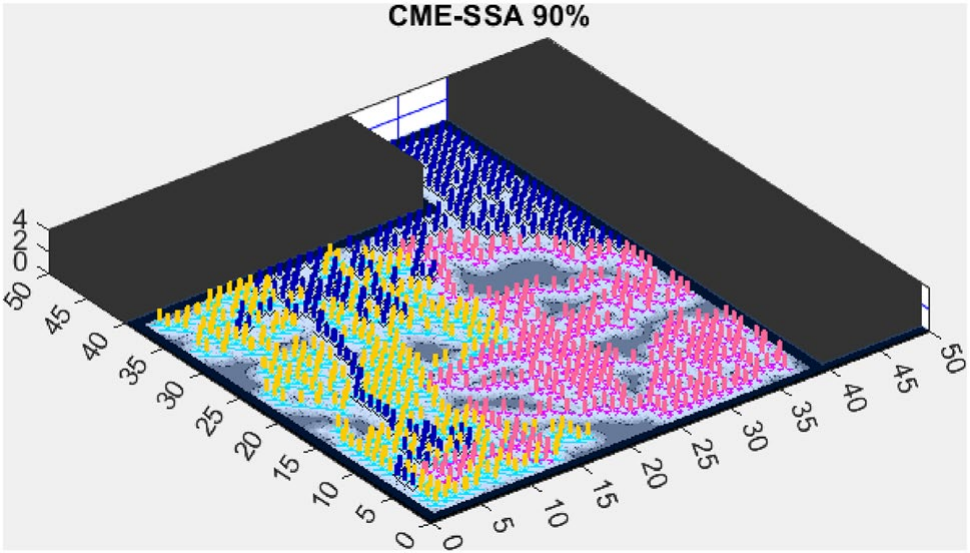
CME-SSA 90%.

### Exploration efficiency on a simplified map (map 1)

In the simulation conducted on a simplified map (Fig. 3), the proposed AMET showed outstanding exploration capabilities, achieving a 96% exploration rate. This impressive metric not only signifies extensive coverage but also denotes the algorithm’s efficiency in navigating and mapping the environment. The blend of deterministic and stochastic elements within AMET likely played a crucial role in its ability to perform a comprehensive sweep of the simulated terrain.

AMET’s effectiveness is evident when compared to other state-of-the-art exploration algorithms. The Coordinated Multi-Robot Exploration and Multi-Objective Grey Wolf Optimizer (CME-MGWO) secured an 89% exploration rate (Fig. 4). Despite being relatively effective, the Grey Wolf Optimizer falls short compared to AMET’s adaptive mechanisms, which seem to be better suited for the exploration tasks at hand. The Coordinated Multi-Robot Exploration and Multi-Objective Ant Colony Optimization (CME-MACO) algorithm showed a lower exploration rate of 79% (Fig. 5). This outcome indicates that the ant foraging behavior, which CME-MACO is modeled after, may have limitations in an environment that does not exhibit the complexity and diversity of a natural setting.

In contrast, the Coordinated Multi-Robot Exploration and Multi-Objective Dragonfly Algorithm (CME-MODA) attained an 81% exploration rate (Fig. 6). The slight improvement over CME-MACO points to the potential advantages of the dragonfly’s swarming behavior in such simplified scenarios. Lastly, the Coordinated Multi-Robot Exploration and Salp Swarm Algorithm (CME-SSA) achieved a 90% exploration rate (Fig. 7). While this is commendable, it underscores the incremental advancements that AMET offers over the baseline salp swarm behavior, enhancing overall exploration efficiency.

The exploration outcomes on the simplified map are indicative of the sophisticated balance AMET strikes between rapid area coverage and detailed environmental assessment. The high exploration rate by AMET suggests a strategic execution that is both swift and methodical—a key attribute for algorithms intended for real-world application. AMET’s superior performance can be attributed to its dynamic adaptation capabilities, which are critical when facing varied and unstructured environments. It is also important to acknowledge the computational aspects of these algorithms. The simplified map context served as a benchmark for assessing the intrinsic exploration capabilities of each algorithm, devoid of external complexities. These results warrant further in-depth exploration into the specific mechanics and decision-making processes embedded within AMET, which may reveal insights for future algorithmic enhancements.

Building on the findings from the first simplified map, we further assessed AMET’s performance on another simplified environment to validate the consistency of its exploration efficiency. While the previous results demonstrated AMET’s ability to achieve high coverage rates in a structured setting, this additional test aims to examine whether similar advantages persist across different simplified terrains. The following section presents the outcomes of this evaluation, comparing AMET with alternative exploration strategies under slightly varied conditions.

**Figure 8:**
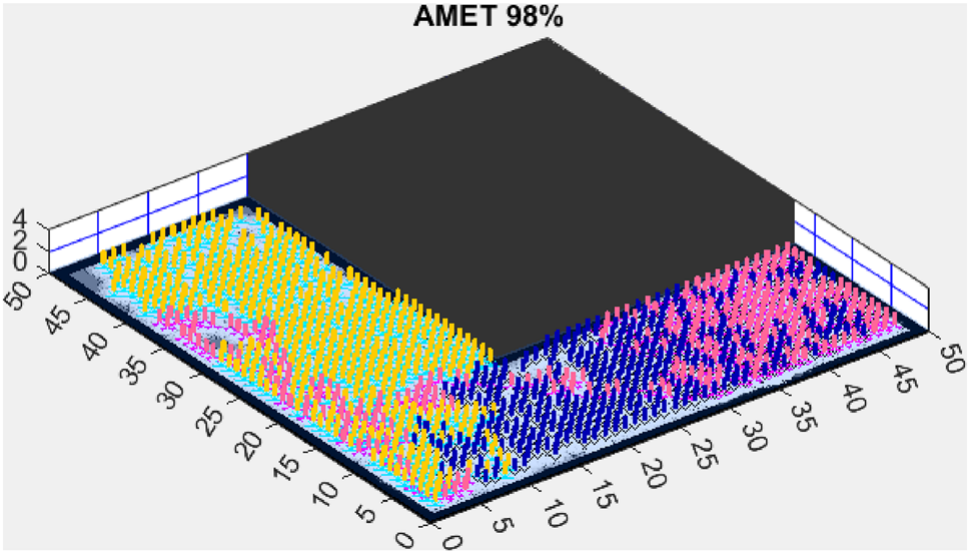
AMET 98% exploration rate.

**Figure 9:**
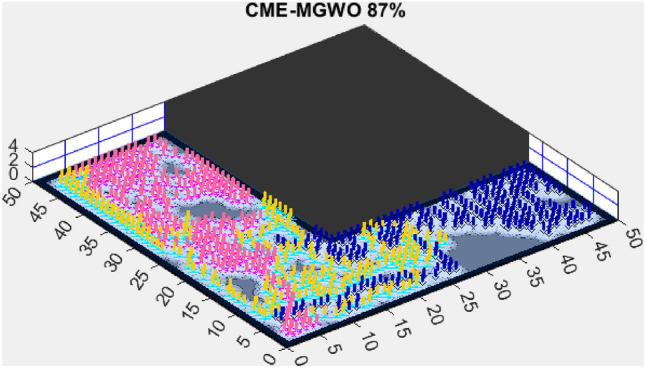
CME-MGWO 87%.

**Figure 10:**
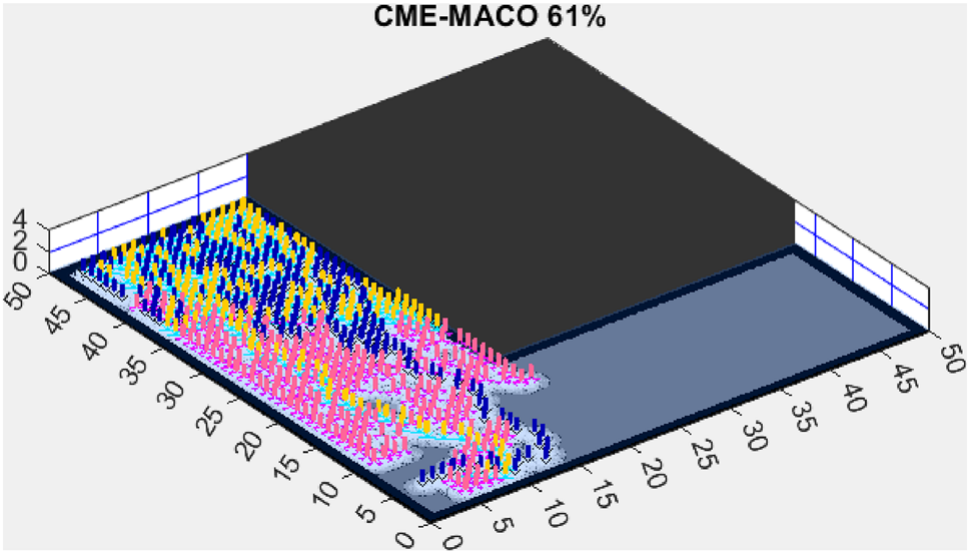
CME-MACO 61%.

**Figure 11:**
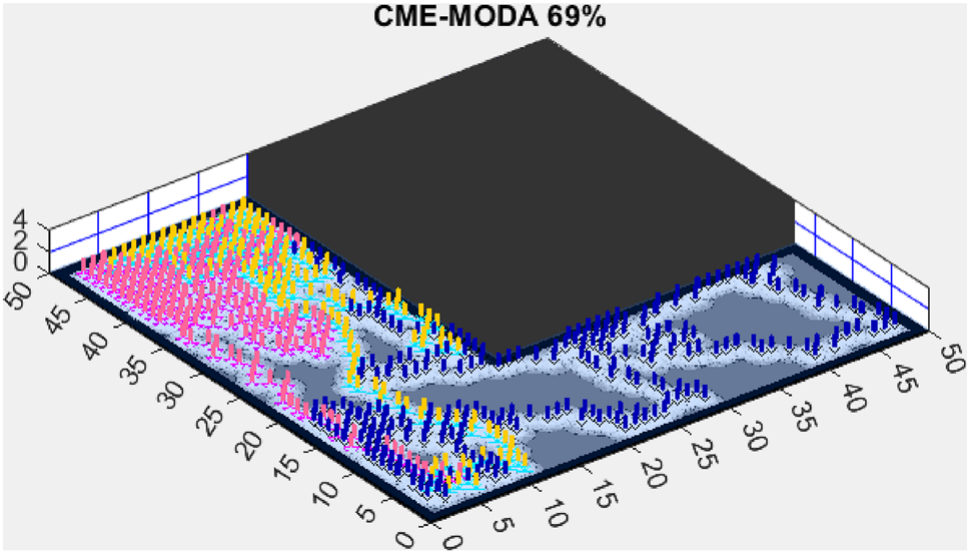
CME-MODA 69%.

**Figure 12:**
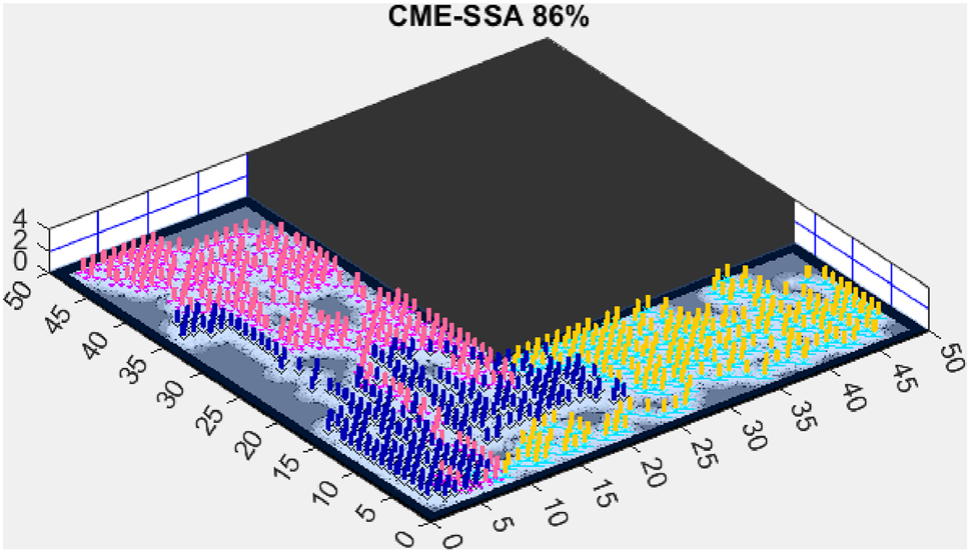
CME-SSA 86%.

### Exploration efficiency on another simplified map (map 2)

The Advanced Multi-Objective Salp Swarm Algorithm Exploration Technique (AMET) demonstrated exceptional performance on the second simplified map, achieving a 98% exploration rate (Fig. 8). This result reinforces AMET’s efficiency in relatively simple environments, where its multi-objective optimization framework effectively balances exploration efficiency and mapping accuracy. The ability to achieve near-complete coverage in such environments highlights AMET’s strong adaptability and decision-making process, ensuring that the robots explore the map optimally while minimizing redundant movements.

A comparison with CME-MGWO, another multi-objective exploration algorithm, shows that while it performed well, achieving an 87% exploration rate (Fig. 9), it fell short of AMET’s results. This suggests that AMET’s more refined balance of multiple objectives plays a crucial role in its superior adaptability. While CME-MGWO successfully navigated the environment, its performance lagged due to its optimization strategy not being as effectively tailored for rapid environmental coverage as AMET’s.

The Coordinated Multi-Robot Exploration and Multi-Objective Ant Colony Optimization (CME-MACO) algorithm, however, exhibited a noticeable drop in efficiency, achieving only 61% exploration (Fig. 10). This significant decrease suggests that CME-MACO’s optimization approach lacks the robustness needed to maintain high performance across different map structures. The variability in environmental complexity appears to have negatively impacted its adaptive decision-making, reducing its ability to efficiently allocate robots for full coverage.

Another tested algorithm, CME-MODA, achieved a 69% exploration rate (Fig. 11). While this performance was better than CME-MACO, it still did not match AMET’s efficiency. CME-MODA’s approach to exploration appears to be partially effective in adapting to simple maps, but its lower optimization resilience compared to AMET suggests that it does not fully account for dynamic changes in the exploration space.

When compared to single-objective methods, the Coordinated Multi-Robot Exploration and Salp Swarm Algorithm (CME-SSA) achieved a notable 86% exploration rate (Fig. 12). Despite being a single-objective algorithm, its performance was comparable to CME-MGWO and surpassed CME-MODA and CME-MACO. This result highlights that well-designed single-objective heuristics can still yield competitive results, particularly when applied to relatively simple environments. However, CME-SSA still lacked the adaptive multi-objective balancing mechanisms present in AMET, which ultimately contributed to AMET’s superior performance.

These results reinforce the importance of multi-objective optimization in exploration tasks, with AMET outperforming both multi-objective and single-objective alternatives in simplified environments. The ability to simultaneously optimize multiple exploration criteria allows AMET to achieve higher exploration rates and maintain consistent performance, making it more effective in adapting to various environmental conditions. The limitations observed in CME-MACO and CME-MODA suggest that not all multi-objective algorithms possess the same level of adaptability, emphasizing the need for sophisticated optimization mechanisms to handle variations in map structure. Meanwhile, the competitive performance of CME-SSA highlights that single-objective approaches can still perform well in relatively simple environments, provided they incorporate effective exploration heuristics.

While the performance on individual maps provides valuable insights into the effectiveness of each algorithm, a broader numerical comparison is necessary to quantify their consistency and efficiency across multiple test scenarios. To further quantify these findings, a numerical comparison of optimization algorithms in simple environments is presented in the next section. This comparison provides a detailed performance breakdown, evaluating key metrics such as exploration efficiency, computational runtime, and task completion rates. Through statistical analysis, we aim to validate the observed trends and further establish the advantages of AMET in multi-robot exploration scenarios.

### Numerical comparison of optimization algorithms in simple environments

Table 1 presents the average explored area and the corresponding standard deviation for various optimization algorithms across two simple environment maps. It is designed to illustrate the comparative performance and consistency of these algorithms in standardized conditions. The average explored area, as shown in Table 1, indicates that the AMET algorithm outperforms the other algorithms in both Map 1 and Map 2, with averages of 94.99% and 95.22% respectively. This superior performance is consistent across the two maps, highlighting the algorithm’s effectiveness in the exploration of simple environments. In terms of stability, as reflected by the standard deviation, the AMET algorithm also leads with lower values (2.29 for Map 1 and 2.51 for Map 2), suggesting a more consistent exploration performance. This consistency is crucial for reliable operation in practical applications, as it implies that AMET is less likely to produce outlier results that deviate from the average performance.

**Table 1:** Average and standard deviation of explored area by optimization algorithms in simple environments. Values in bold indicate the best performance for each metric (highest average explored area or lowest standard deviation).

Simple map	Algorithm	Average explored area (%)	Standard deviation
Map 1	AMET	**94.99**	**2.29**
	CME-MGWO	89.47	3.01
	CME-SSA	89.90	2.97
	CME-MODA	82.88	8.95
	CME-MACO	86.95	6.16
Map 2	AMET	**95.22**	**2.51**
	CME-MGWO	91.02	3.49
	CME-SSA	91.84	3.10
	CME-MODA	84.75	6.99
	CME-MACO	87.46	8.01

Comparatively, the CME-MODA and CME-MACO algorithms exhibit higher standard deviations, especially in Map 1, with values of 8.95 and 6.16 respectively, which indicates less consistency in their exploration results. Higher standard deviations point towards a greater variability in the explored area from one simulation to another, which can be a disadvantage in scenarios where reliability is key. This analysis underscores the importance of both high average performance and low variability in the selection of an optimization algorithm for practical exploration tasks. The AMET algorithm’s combination of high average explored area and low standard deviation across simple maps positions it as a preferable choice for tasks requiring efficient and consistent area coverage.

Table 2 summarizes the average time taken (in seconds) by various optimization algorithms to complete 500 iterations in Simple Map 1 and Simple Map 2, along with the corresponding standard deviations. The analysis of the average time, as shown in Table 2, indicates that all algorithms are relatively efficient, with AMET demonstrating the quickest average completion time in Simple Map 1 at 93.98 seconds. However, in Simple Map 2, AMET’s average completion time is marginally higher at 94.32 seconds, yet still under the 95-second mark, which indicates a high level of time efficiency in both maps. Notably, the standard deviation values for AMET are the lowest in Map 1, demonstrating its consistent time performance across runs. This consistency is slightly less in Map 2, as indicated by a slight increase in the standard deviation; however, it remains relatively low, underscoring AMET’s stability in time efficiency.

**Table 2:** Average time and standard deviation for completion of 500 iterations by optimization algorithms in simple environments. Values in bold indicate the best performance for each metric (lowest average time and lowest standard deviation).

Simple map	Algorithm	Average time (s)	Standard deviation
Map 1	AMET	**93.98**	**0.30**
	CME-MGWO	98.91	1.00
	CME-SSA	97.38	0.18
	CME-MODA	99.34	1.23
	CME-MACO	98.30	1.02
Map 2	AMET	**94.32**	**0.40**
	CME-MGWO	97.99	1.01
	CME-SSA	97.69	0.29
	CME-MODA	99.59	0.90
	CME-MACO	99.99	1.14

In comparison, CME-MODA and CME-MACO have the highest average times in both maps, with standard deviations that suggest greater variability in their time efficiency. This could indicate that while these algorithms may perform adequately on average, their performance can vary more significantly from run to run. The findings from this table indicate that while the differences in average completion times between the algorithms are relatively minor, the consistency of performance—as evidenced by the standard deviation—is an important factor. AMET’s stable and efficient performance in terms of time across multiple runs makes it a reliable option for tasks where time efficiency is critical.

Table 3 outlines the number of times each optimization algorithm failed to continue and complete a simulation across two simple maps. These failures can indicate the robustness and reliability of each algorithm under standard operating conditions. In Simple Map 1, AMET and CME-SSA show no instances of simulation failure, demonstrating their robustness and the reliability of their exploration strategies. In contrast, CME-MGWO, CME-MODA, and CME-MACO have reported failures, with CME-MGWO and CME-MODA showing a higher count at 6 failed simulations each, and CME-MACO at 5. Simple Map 2 shows a similar trend with AMET reporting no failures once again, underscoring its consistency and resilience. CME-SSA experiences a slight increase in failures to 1, while CME-MGWO’s failures decrease to 2. Notably, CME-MODA’s and CME-MACO’s failed simulations increase to 9 and 8, respectively, suggesting potential issues with their stability or adaptability in this environment.

**Table 3:** Number of failed simulations by optimization algorithms in simple environments. Bold values indicate the best performance, i.e., the lowest number of failed simulations, reflecting higher reliability.

Simple map	Algorithm	Number of failed simulations	
Map 1	AMET	**0**	
	CME-MGWO	6	
	CME-SSA	**0**	
	CME-MODA	6	
	CME-MACO	5	
Map 2	AMET	**0**	
	CME-MGWO	2	
	CME-SSA	1	
	CME-MODA	9	
	CME-MACO	8	

The absence of failures for AMET across both maps indicates a significant level of dependability, an essential attribute for algorithms intended for real-world autonomous exploration tasks. The results suggest that AMET can consistently perform without interruptions or issues that lead to simulation failure. In comparison, the increased number of failures for other algorithms, particularly CME-MODA and CME-MACO on Map 2, points to possible areas for improvement in their operational robustness. These failures can have implications for the practical application of these algorithms, as they may result in incomplete exploration tasks or require additional resources to manage and mitigate simulation failures.

While the numerical comparison in simple environments provides strong evidence of AMET’s superiority in terms of exploration efficiency, stability, and computational performance, real-world applications often involve more complex and dynamic environments. To assess the robustness of AMET and its competitors in more challenging conditions, we extend the evaluation to complex maps. The following section presents the exploration efficiency of the tested algorithms in a structured yet obstacle-rich environment, analyzing their adaptability and performance under increased complexity.

**Figure 13:**
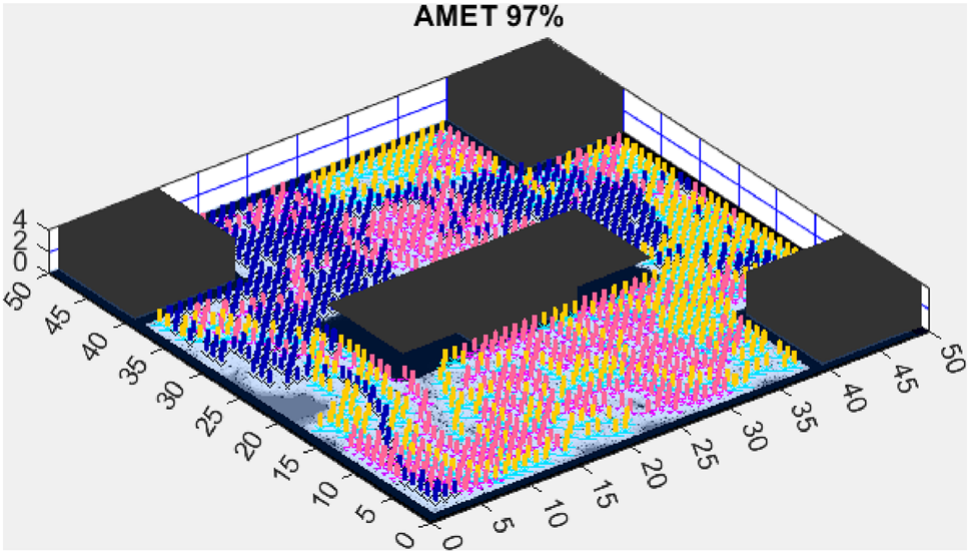
AMET 97% exploration rate.

**Figure 14:**
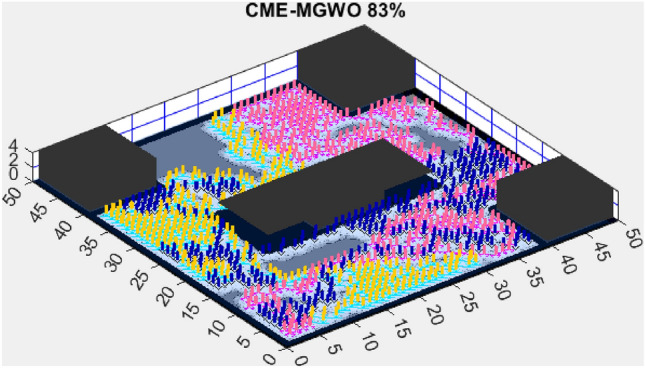
CME-MGWO 83%.

**Figure 15:**
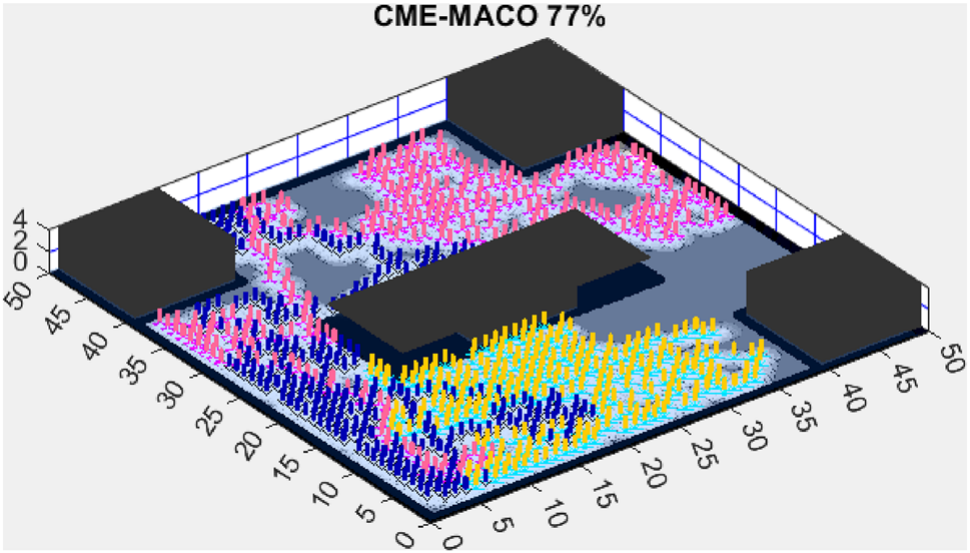
CME-MACO 77%.

**Figure 16:**
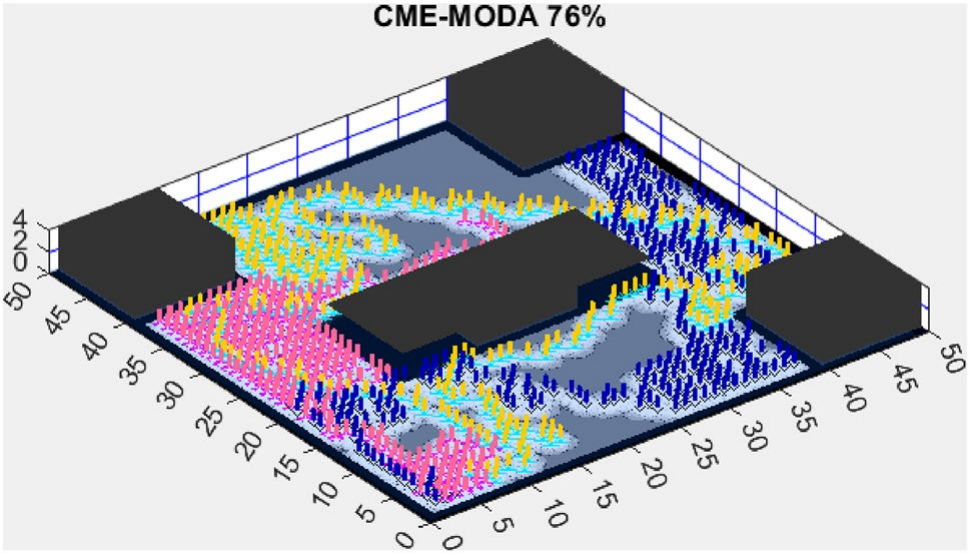
CME-MODA 76%.

**Figure 17:**
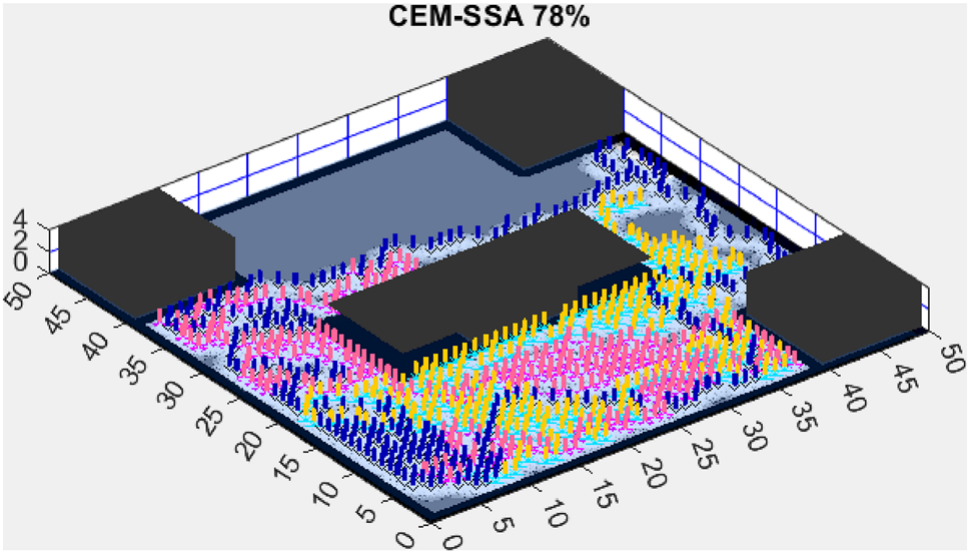
CME-SSA 78%.

### Exploration efficiency on complex map 1

On our first complex map, AMET demonstrated a slightly reduced yet still exceptional 97% exploration rate (Fig. 13). This slight reduction from the simplified maps may be attributed to the increased environmental obstacles and complexity, challenging the algorithm’s pathfinding capabilities. However, AMET’s high adaptability and robust multi-objective optimization allowed for near-complete coverage of the complex terrain. The performance of the multi-objective algorithms on Complex Map 1 exhibited a noticeable decline compared to their success on the simplified maps. CME-MGWO experienced a substantial decrease to an 83% exploration rate (Fig. 14), suggesting that the algorithm may require further refinement to maintain its efficiency in more challenging environments.

CME-MACO’s exploration rate on Complex Map 1 was 77% (Fig. 15), which is a significant drop from the performance on the simplified maps. This result reinforces the notion that the ant colony optimization strategies might struggle with the navigation and decision-making required in complex scenarios. Similarly, CME-MODA recorded a 76% exploration rate (Fig. 16). While this indicates that the algorithm can still navigate through complex environments to some extent, it also suggests that the dragonfly-inspired optimization may not be entirely suited for highly intricate terrains. CME-SSA achieved a 78% exploration rate (Fig. 17), which is noteworthy considering its design for optimizing a single criterion. This performance highlights the effectiveness of the salp swarm’s heuristic approach even when faced with complex environmental structures.

The results from Complex Map 1 clearly illustrate the importance of sophisticated optimization strategies in multi-robot exploration algorithms. AMET’s minimal performance decrease showcases the effectiveness of its multi-objective approach, which appears to be more resilient to increased complexity compared to its counterparts. The decline in exploration rates among the other algorithms suggests that complex environments require more than just the direct application of bio-inspired heuristics; they demand adaptive strategies capable of handling diverse and unpredictable variables. This is where AMET’s multi-objective optimization shines, balancing exploration efficiency and mapping accuracy even under challenging conditions. These findings suggest that AMET could be a viable solution for real-world applications, where environments are often complex and unpredictable. Its ability to maintain high performance in such settings could be particularly beneficial for tasks like search and rescue operations, environmental monitoring, and other scenarios requiring comprehensive area coverage.

While the first complex map provided valuable insights into how AMET and its counterparts handle increased environmental difficulty, further evaluation is necessary to confirm the consistency of these trends. To strengthen the analysis, we assess the performance of the algorithms on a second complex map with different structural characteristics. This additional test allows for a more comprehensive understanding of each algorithm’s adaptability, robustness, and efficiency under varying complex conditions.

**Figure 18:**
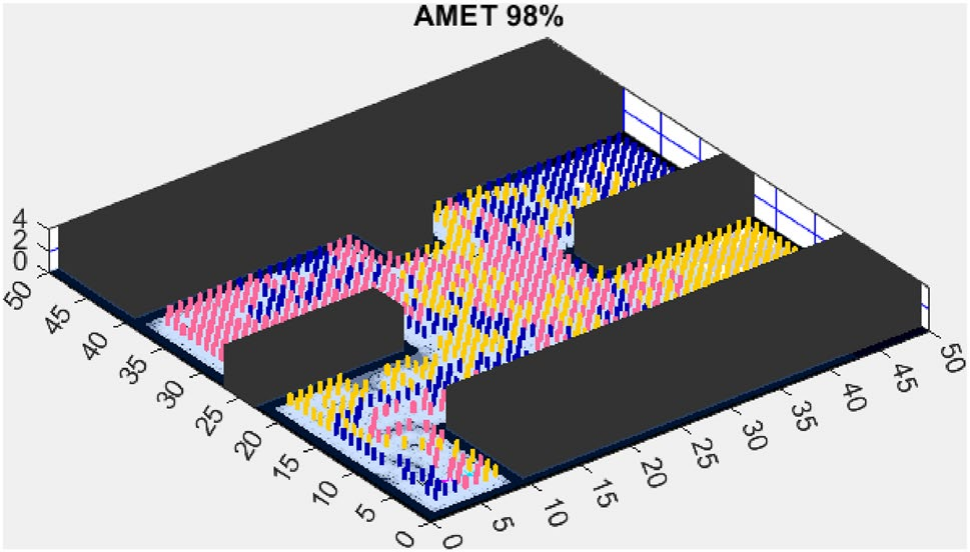
AMET 98% exploration rate.

**Figure 19:**
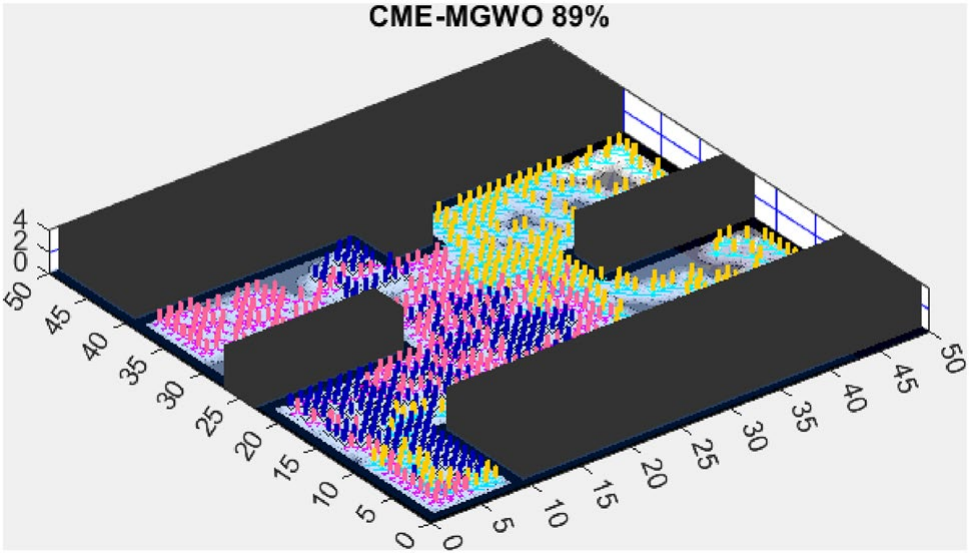
CME-MGWO 89%.

**Figure 20:**
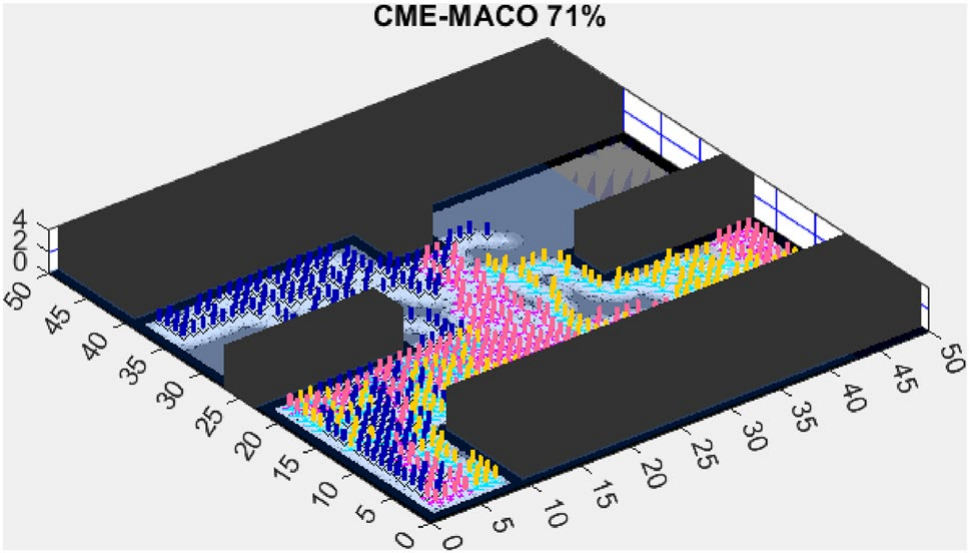
CME-MACO 71%.

**Figure 21:**
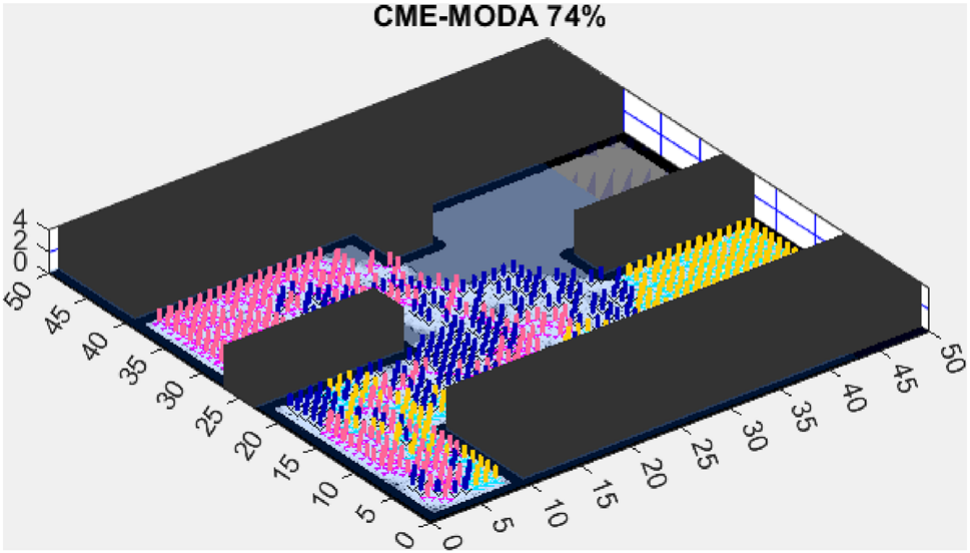
CME-MODA 74%.

**Figure 22:**
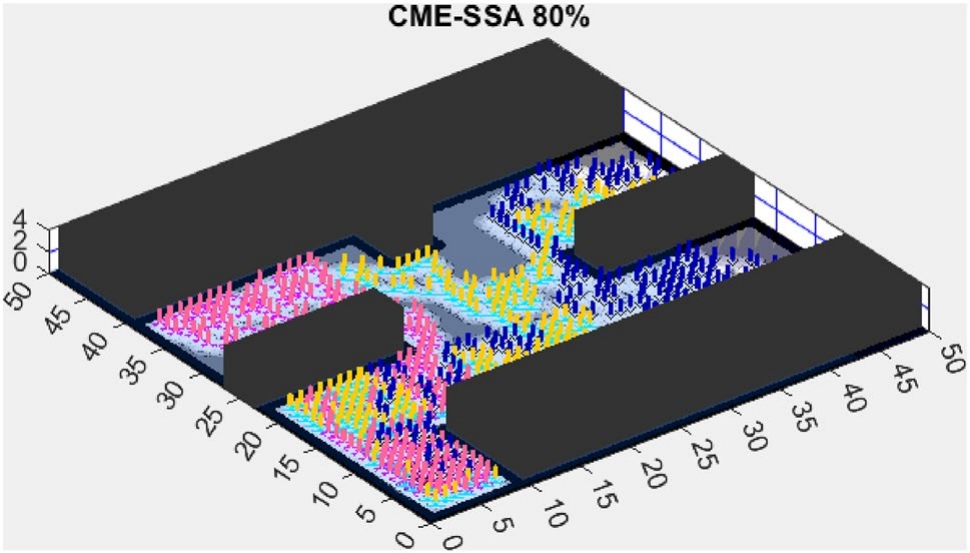
CME-SSA 80%.

### Exploration efficiency on complex map 2

In the second complex environment, the proposed AMET sustained its high performance, achieving a 98% exploration rate (Fig. 18). This remarkable result is indicative of AMET’s robustness and its ability to navigate complex environments efficiently, improving upon its already impressive performance in the first complex map. CME-MGWO showed a significant improvement on Complex Map 2 with an 89% exploration rate (Fig. 19). This improvement suggests that CME-MGWO’s strategies may be more suited to the specific challenges presented by this map, or it may have benefited from the unique distribution of obstacles and open areas in this particular environment.

CME-MACO struggled on Complex Map 2, with the exploration rate dropping to 71% (Fig. 20). This decrease further highlights the challenges that ant colony optimization algorithms face in adapting to complex environments where direct pheromone-following behavior may not suffice. CME-MODA managed only a 74% exploration rate (Fig. 21). While slightly better than its performance on the first complex map, it still underscores the need for more adaptive and nuanced navigation strategies to cope with complex terrain. CME-SSA presented an 80% exploration rate (Fig. 22), demonstrating a notable improvement from the first complex map. This performance showcases the strengths of the salp swarm heuristic in certain complex scenarios, even as a single-objective algorithm.

AMET’s consistently high performance across different complex maps is a testament to the algorithm’s advanced design, which allows for effective multi-objective optimization. Its ability to adapt to the complexity of the environment, without a significant drop in exploration efficiency, suggests that AMET can handle a variety of real-world scenarios where unpredictability and complexity are the norms. The varied performances of the other algorithms indicate that the specific features of a complex environment can significantly impact the effectiveness of exploration strategies. The improvements seen in CME-MGWO and CME-SSA suggest that certain map configurations may play to the strengths of their respective heuristic approaches. These results emphasize the necessity of versatile and dynamic exploration strategies in the field of autonomous robotic systems. They highlight the potential of algorithms like AMET in leading the way for future developments in this domain.

The results from Complex Map 2 further validate AMET’s superior adaptability in challenging environments. While CME-MGWO and CME-SSA showed improvements in performance, other algorithms continued to struggle with maintaining high exploration efficiency. To reinforce these findings and examine how AMET and its counterparts handle even greater complexity, we extend our evaluation to a third complex map. This next analysis provides deeper insights into the scalability and robustness of each algorithm under increasingly difficult exploration conditions.

**Figure 23:**
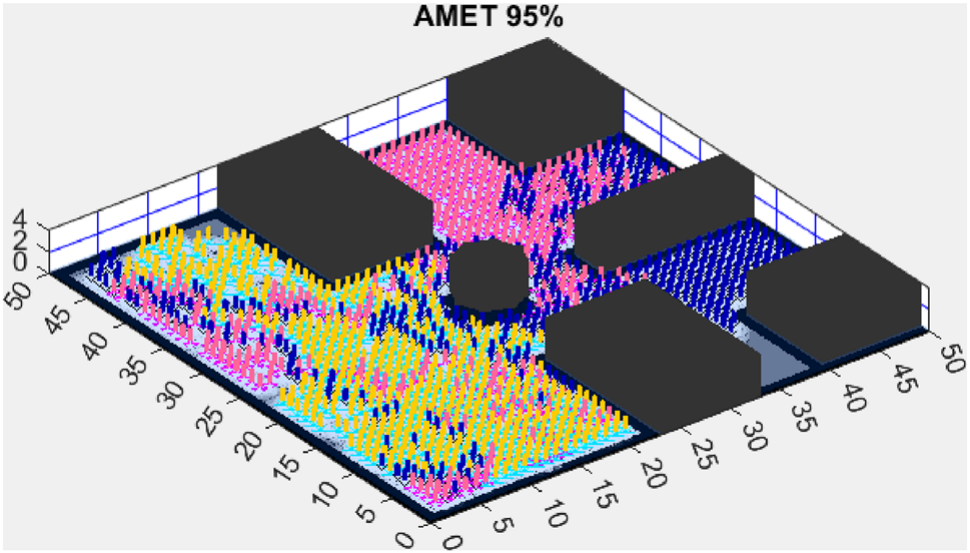
AMET 95% exploration rate.

**Figure 24:**
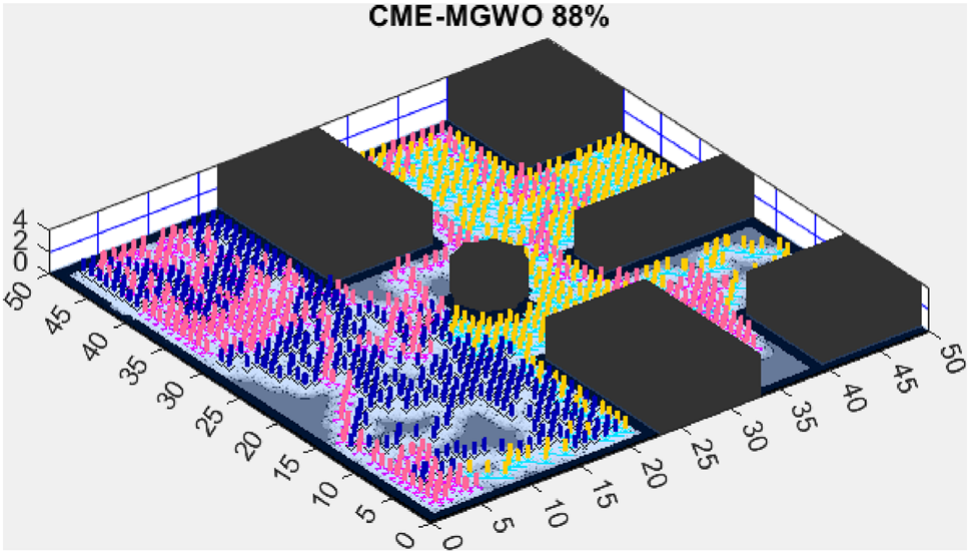
CME-MGWO 88%.

**Figure 25:**
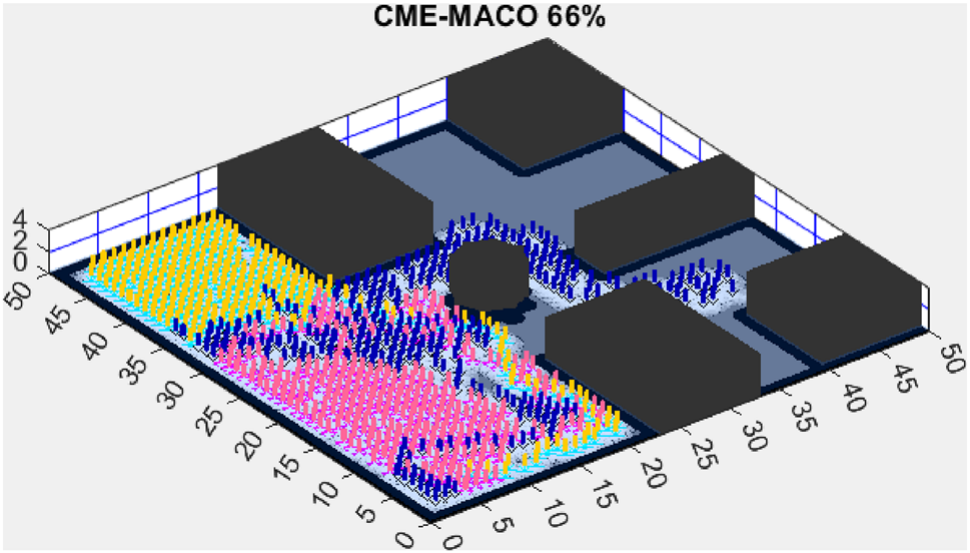
CME-MACO 66%.

**Figure 26:**
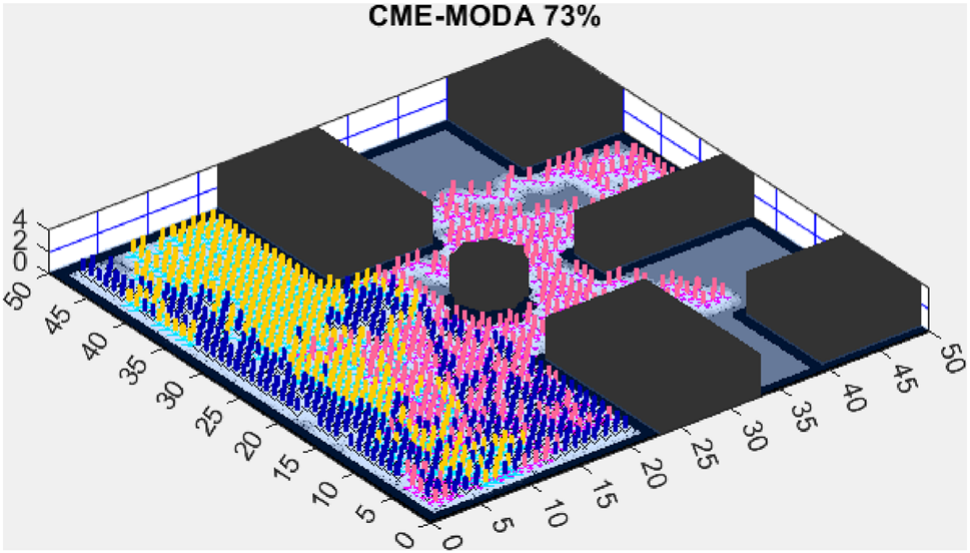
CME-MODA 73%.

**Figure 27:**
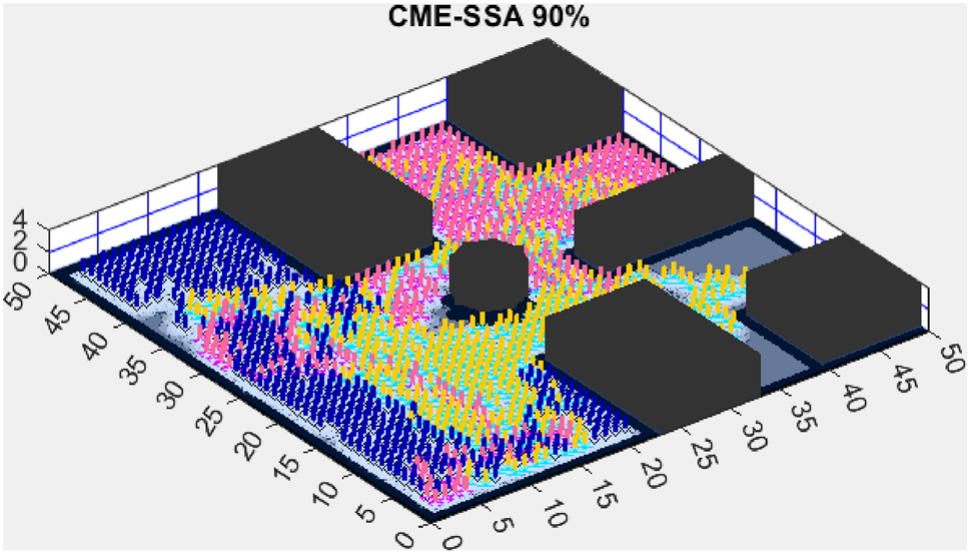
CME-SSA 90%.

### Exploration efficiency on complex map 3

On Complex Map 3, AMET showcased a slight decrease in performance with a 95% exploration rate (Fig. 23). Despite this being the lowest performance observed for AMET across all maps, it remains remarkably high, affirming the algorithm’s adaptability and robust multi-objective optimization in handling complex scenarios. CME-MGWO maintained a strong performance with an 88% exploration rate (Fig. 24), indicating that its optimization strategies, inspired by the social hierarchy and hunting techniques of grey wolves, are relatively effective in complex environments, albeit less so than AMET. CME-MACO algorithm experienced a considerable drop in its exploration rate to 66% (Fig. 25). This reduction reinforces the challenges faced by ant colony optimization in navigating environments with increased complexity, possibly due to a reliance on path pheromones that may become less efficient as complexity escalates.

CME-MODA recorded a 73% exploration rate (Fig. 26), showing that while the dragonfly-inspired algorithm has some capability to adapt to complex environments, it still struggles to achieve the level of performance seen in AMET. Remarkably, CME-SSA reached a 90% exploration rate (Fig. 27), displaying significant resilience on Complex Map 3. This suggests that the single-objective focus of CME-SSA on exploration efficiency might be beneficial in certain complex scenarios, although it does not consistently outperform the multi-objective AMET.

The trends observed on Complex Map 3 are consistent with previous findings, highlighting the superior adaptability of AMET in various environmental settings. The slight decline in AMET’s performance, while noteworthy, does not detract from its overall effectiveness as a top-performing algorithm in complex exploration tasks. The fluctuating performances of the other algorithms underscore the influence of environmental features on the efficiency of exploration strategies. It is evident that the sophistication and adaptability of AMET’s multi-objective optimization are key to its success, allowing it to outperform others even as the complexity of the map increases.

The results from Complex Map 3 further reinforce AMET’s ability to maintain high exploration efficiency, even as environmental complexity increases. While CME-SSA demonstrated notable resilience, other algorithms continued to struggle with adaptability and navigation. To assess whether these trends persist across different complex scenarios, we proceed with an evaluation on Complex Map 4. This next analysis provides deeper insights into the consistency and robustness of each algorithm when faced with varying obstacle distributions and terrain intricacies.

**Figure 28:**
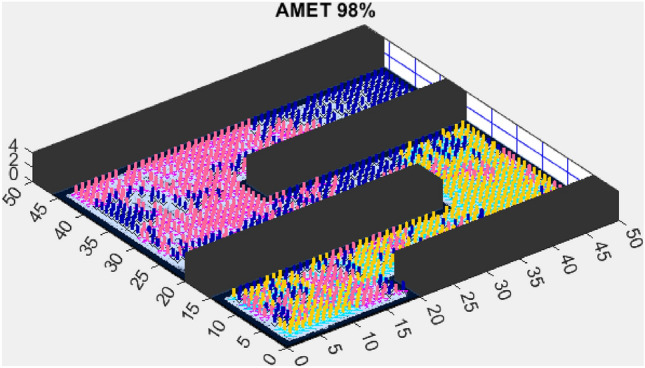
AMET 98% exploration rate.

**Figure 29:**
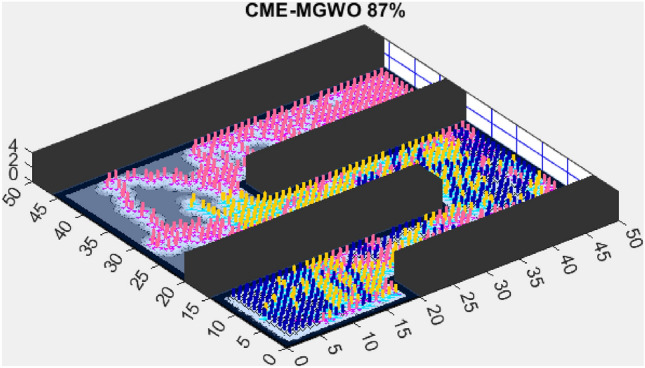
CME-MGWO 87%.

**Figure 30:**
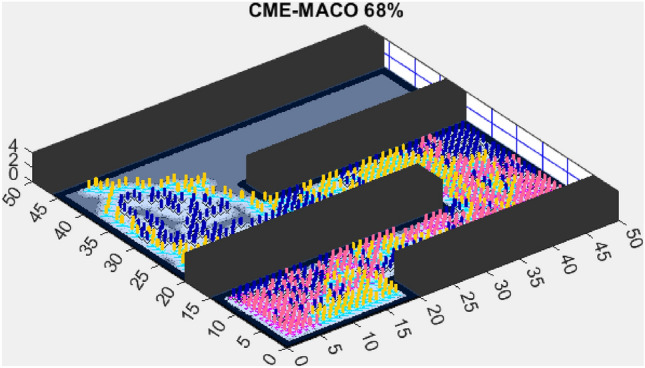
CME-MACO 68%.

**Figure 31:**
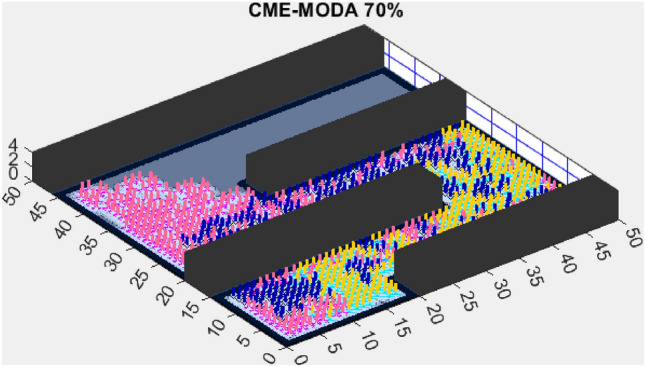
CME-MODA 70%.

**Figure 32:**
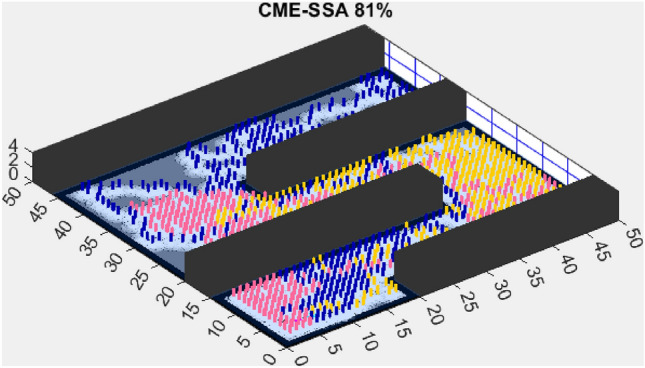
CME-SSA 81%.

### Exploration efficiency on complex map 4

Once again, the proposedAMET demonstrates a high exploration rate, this time achieving 98% on Complex Map 4 (Fig. 28). This result is consistent with the algorithm’s previous performances, showcasing its robustness and reinforcing its suitability for complex environments. CME-MGWO maintained a respectable 87% exploration rate (Fig. 29), suggesting that while the algorithm is effective, it may not be as adept at dealing with the intricacies of more complex environments as AMET. CME-MACO showed a significant challenge on Complex Map 4 with a 68% exploration rate (Fig. 30), which might indicate limitations in its algorithmic structure when it comes to adapting to the spatial complexity and obstacle density present in this environment.

CME-MODA achieved a 70% exploration rate (Fig. 31). While it showed some improvement over CME-MACO, it still falls short of the high benchmark set by AMET, suggesting that further enhancements are needed for CME-MODA to navigate complex terrains effectively. CME-SSA recorded an 81% exploration rate (Fig. 32). As a single-objective algorithm, CME-SSA continues to exhibit commendable performance, though it does not reach the high efficiency demonstrated by the multi-objective AMET.

The results from Complex Map 4 continue to highlight AMET’s advanced capabilities in adaptive exploration. Its consistent performance across various complex maps is indicative of a well-designed algorithm capable of handling diverse and challenging environments. The other algorithms show varied results, with some like CME-MGWO and CME-SSA performing relatively well, while others such as CME-MACO and CME-MODA struggle with the increased complexity. These differences underscore the importance of multi-objective optimization in exploration tasks and the potential limitations of single-objective and less adaptive multi-objective algorithms in certain scenarios.

The results from Complex Map 4 reaffirm AMET’s capability to sustain high exploration efficiency across different complex environments. While CME-SSA and CME-MGWO demonstrated moderate adaptability, other algorithms struggled significantly with navigation and coverage. To further examine AMET’s robustness under increasingly challenging conditions, we extend our analysis to Complex Map 5. This final evaluation will assess how well each algorithm copes with an even greater degree of environmental complexity, providing a comprehensive understanding of their scalability and real-world applicability.

**Figure 33:**
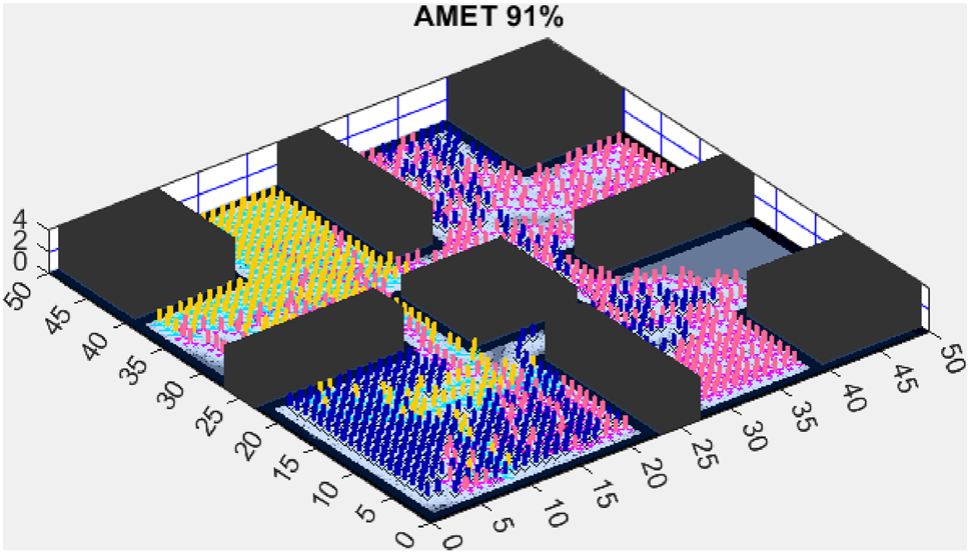
AMET 91% exploration rate.

**Figure 34:**
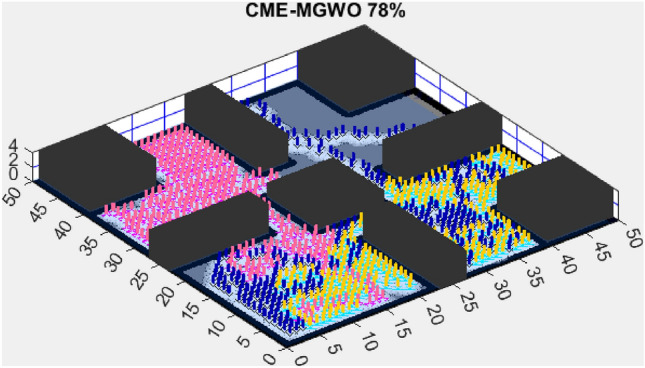
CME-MGWO 78%.

**Figure 35:**
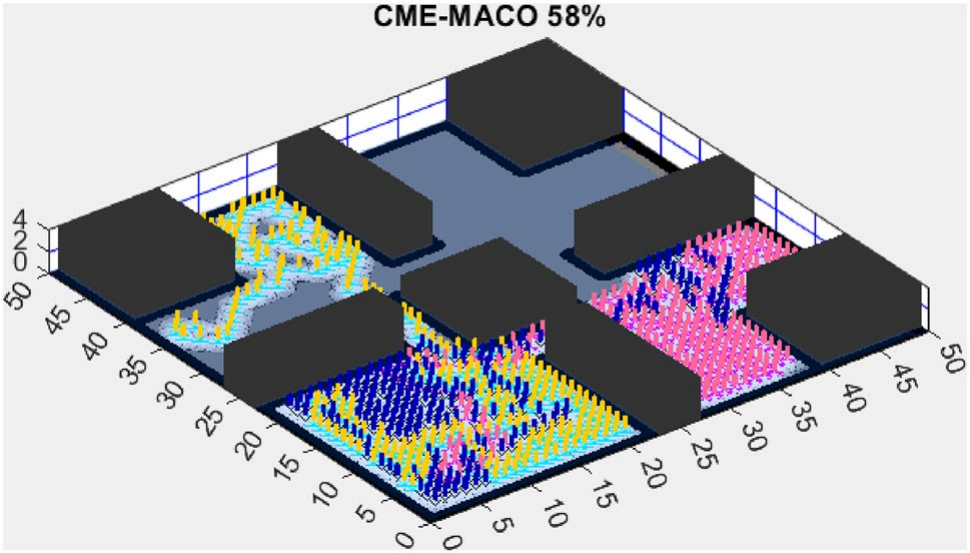
CME-MACO 58%.

**Figure 36:**
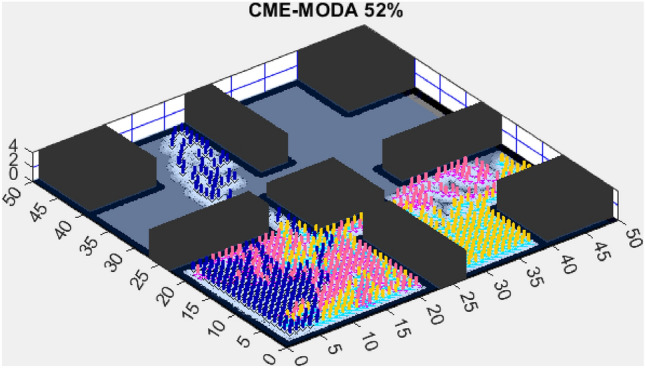
CME-MODA 52%.

**Figure 37:**
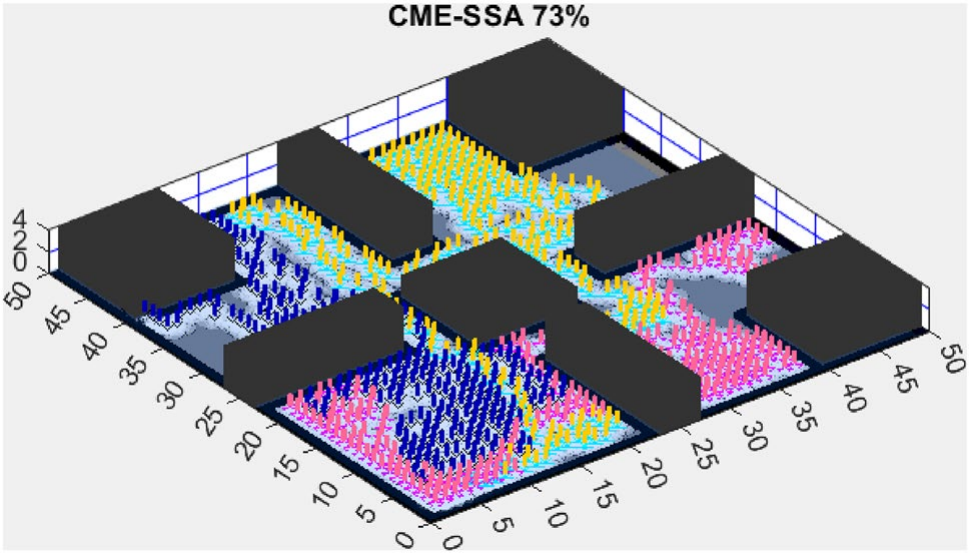
CME-SSA 73%.

### Exploration efficiency on complex map 5

On Complex Map 5, the Advanced Multi-Objective Salp Swarm Algorithm Exploration Technique (AMET) achieved a 91% exploration rate (Fig. 33). This performance, while slightly lower than on previous maps, still showcases the algorithm’s proficiency in navigating and mapping complex environments effectively. CME-MGWO experienced a decrease to a 78% exploration rate (Fig. 34). This performance drop might reflect the algorithm’s limitations in adapting to the particular challenges posed by the complex structure of Map 5. CME-MACO’s exploration rate further declined to 58% on Complex Map 5 (Fig. 35). The complexity and the intricate layout of this map likely exacerbated the limitations of the ant colony optimization principles, especially in areas with dense obstacles or limited pheromone trails.

The CME-MODA algorithm recorded a significant drop to a 52% exploration rate (Fig. 36). This marked decrease suggests that the navigation and alignment mechanisms inherent to the dragonfly algorithm might not be sufficient for highly complex environments that require more flexible and adaptable exploration strategies. CME-SSA achieved a 73% exploration rate (Fig. 37), demonstrating that it can still perform reasonably well despite the challenges of the environment, though it does not consistently reach the efficiency of AMET.

Table 4 displays the average percentage of the explored area and the corresponding standard deviations for each optimization algorithm across five complex maps. This data provides insight into how well each algorithm performs in terms of coverage in more challenging and intricate environments. Analyzing the average explored area percentages, as shown in Table 4, AMET consistently achieves the highest coverage across all five complex maps, with its lowest average still being an impressive 95.00% on Map 5. This consistent top performance demonstrates AMET’s superior exploration capabilities in complex environments, attributable to its multi-objective optimization framework that effectively balances the exploration of new areas with the accuracy of the mapping.

**Table 4:** Explored area performance by optimization algorithms in complex environments. Bold values indicate the best performance for each metric—highest average explored area or lowest standard deviation.

Complex map	Algorithm	Average explored area (%)	Standard deviation
Map 1	AMET	**95.88**	**2.41**
	CME-MGWO	88.99	3.78
	CME-SSA	91.55	4.22
	CME-MODA	86.65	8.85
	CME-MACO	87.51	9.46
Map 2	AMET	**96.57**	**1.97**
	CME-MGWO	90.85	5.63
	CME-SSA	92.46	4.42
	CME-MODA	86.65	9.96
	CME-MACO	86.87	7.89
Map 3	AMET	**97.87**	**2.44**
	CME-MGWO	92.00	4.85
	CME-SSA	95.90	3.24
	CME-MODA	88.99	9.25
	CME-MACO	85.12	8.32
Map 4	AMET	**97.90**	**3.31**
	CME-MGWO	87.75	7.45
	CME-SSA	88.88	7.96
	CME-MODA	79.14	13.53
	CME-MACO	79.54	14.52
Map 5	AMET	**95.00**	**2.90**
	CME-MGWO	88.25	7.95
	CME-SSA	84.46	8.75
	CME-MODA	65.22	15.26
	CME-MACO	74.25	16.21

When examining the standard deviation, AMET maintains lower variability in the explored area, indicating stable and reliable performance across different runs. Its standard deviation is notably smaller than that of the other algorithms, especially in Maps 4 and 5, where the complexity of the environment significantly impacts the performance stability of the other algorithms. Other algorithms such as CME-MGWO, CME-SSA, CME-MODA, and CME-MACO show varying degrees of success, with generally lower average exploration percentages and higher standard deviations. This variability could imply a less consistent performance and a potential for unpredictability in different complex map scenarios. CME-MODA and CME-MACO, in particular, exhibit the highest standard deviations on Maps 4 and 5, signaling considerable fluctuations in their explored areas. This inconsistency could be detrimental in applications where consistent performance is crucial.

Table 5 outlines the average time taken (in seconds) for each optimization algorithm to complete a full run consisting of 500 iterations across five complex maps, along with the corresponding standard deviations. This data reflects the time efficiency of each algorithm in navigating and exploring complex environments.In all five complex maps, as shown in Table 5, AMET consistently demonstrates the shortest average time to complete 500 iterations, indicating its efficiency in terms of time management. The relatively low standard deviation values also highlight AMET’s stability and reliability in maintaining consistent time performance across different runs.

**Table 5:** Time taken for completion of 500 iterations by optimization algorithms in complex environments. Values in bold indicate the best performance for each metric (lowest average time and lowest standard deviation).

Complex map	Algorithm	Average time (s)	Standard deviation
Map 1	AMET	**94.31**	**0.28**
	CME-MGWO	98.78	0.58
	CME-SSA	97.96	0.35
	CME-MODA	99.28	1.08
	CME-MACO	100.07	1.04
Map 2	AMET	**94.80**	**0.15**
	CME-MGWO	98.87	1.01
	CME-SSA	98.03	0.26
	CME-MODA	101.22	1.18
	CME-MACO	100.57	1.84
Map 3	AMET	**94.78**	**0.20**
	CME-MGWO	99.03	2.05
	CME-SSA	98.20	0.51
	CME-MODA	101.93	1.46
	CME-MACO	101.87	1.53
Map 4	AMET	**93.77**	**0.23**
	CME-MGWO	98.88	1.24
	CME-SSA	98.49	0.58
	CME-MODA	102.43	1.25
	CME-MACO	101.68	1.58
Map 5	AMET	**94.46**	**0.28**
	CME-MGWO	98.66	0.75
	CME-SSA	98.48	0.58
	CME-MODA	102.05	1.26
	CME-MACO	102.14	1.68

Comparatively, CME-MODA and CME-MACO exhibit the longest average times, especially noticeable in Maps 4 and 5. Their higher standard deviations suggest a greater variability in time efficiency, potentially impacting their reliability in time-sensitive applications. CME-MGWO and CME-SSA show moderate time efficiency, with CME-MGWO having slightly higher average times and standard deviations than CME-SSA. While these algorithms complete the iterations in reasonable times, they are not as efficient as AMET, particularly in more complex maps where the challenge of navigation and exploration is heightened.

Table 6 details the number of failed simulations for each optimization algorithm across five complex maps. The failure to continue a simulation can indicate the robustness of an algorithm and its suitability for complex environments.

**Table 6:** Number of failed simulations by optimization algorithms in complex environments. Bold values indicate the best performance, i.e., the lowest number of failed simulations, reflecting higher reliability.

Complex map	Algorithm	Number of failed simulations
Map 1	AMET	**0**
	CME-MGWO	55
	CME-SSA	**0**
	CME-MODA	48
	CME-MACO	74
Map 2	AMET	**0**
	CME-MGWO	5
	CME-SSA	**0**
	CME-MODA	15
	CME-MACO	88
Map 3	AMET	**2**
	CME-MGWO	18
	CME-SSA	**2**
	CME-MODA	13
	CME-MACO	28
Map 4	AMET	**1**
	CME-MGWO	101
	CME-SSA	5
	CME-MODA	133
	CME-MACO	54
Map 5	AMET	**3**
	CME-MGWO	99
	CME-SSA	4
	CME-MODA	417
	CME-MACO	103

In all five complex maps, as shown in Table 6, AMET demonstrates exceptional robustness with the lowest number of failed simulations. Specifically, it shows no failures in Maps 1 and 2, and only a few in the remaining maps, highlighting its reliability in complex environments. CME-MGWO, CME-MODA, and CME-MACO exhibit a significant number of failures across the maps, with particularly high counts in Maps 4 and 5. This suggests potential stability issues or limitations in their ability to adapt to the complexities of these environments. Notably, CME-MODA experiences an exceptionally high number of failures in Map 5, indicating a substantial challenge in this specific scenario. CME-SSA shows a generally low number of failures, comparable to AMET, which suggests a degree of robustness. However, its performance still does not match the near-perfect record of AMET.

The results from Complex Map 5 reaffirm AMET’s ability to maintain a high level of exploration efficiency, even in highly intricate environments. While some algorithms, such as CME-SSA and CME-MGWO, demonstrated moderate adaptability, others struggled significantly with navigation and overall coverage. To further validate the statistical significance of these performance differences, we conducted the Wilcoxon rank-sum test. This test provides a rigorous comparative analysis of AMET’s exploration efficiency and time performance against competing algorithms, ensuring that the observed superiority is not due to random variation.

### Wilcoxon statistical test results

To ensure the statistical validity of the observed differences in performance, we conducted the Wilcoxon rank-sum test, a non-parametric statistical test commonly used for comparing two independent distributions. Each algorithm was executed 30 times over 500 iterations, allowing for a comprehensive evaluation of exploration efficiency and time performance. The average exploration rate provides insight into an algorithm’s typical performance, while the standard deviation highlights stability and consistency across multiple runs (referenced in Tables 1, 2, 3, 4, 5 and 6). The Wilcoxon test was used to determine whether AMET’s observed superiority was statistically significant or if it could be attributed to random variation.

To structure the analysis, two hypotheses were established:

- Null Hypothesis ($H_0$): For the chosen performance metric,  AMET is not better than the comparison algorithm.
- Alternative Hypothesis ($H_1$):  AMET outperforms the comparison algorithm.

A *p*-value threshold of 0.05 was used, meaning that values below 0.05 indicate a statistically significant difference, providing evidence to reject the null hypothesis ($H_0$) and confirm that AMET’s superior performance is not due to chance.

For each test, AMET’s performance was compared against the best-performing alternative among CME-MGWO, CME-SSA, CME-MODA, and CME-MACO. This approach ensured a consistent evaluation framework, reinforcing the reliability of the conclusions regarding AMET’s efficacy in multi-robot exploration.

**Table 7:** *p*-values from Wilcoxon rank sum test on exploration data.

Map type	Map No	AMET versus CME-MGWO	AMET versus CME-SSA	AMET versus CME-MODA	AMET versus CME-MACO
Simple	Map 1	0.00020571	0.076	0.0016	0.0254
	Map 2	2.11E−06	3.01E−05	1.62E−08	2.48E−08
Complex	Map 1	0.000121111	2.02E−04	6.48E−05	3.58E−05
	Map 2	6.09E−08	3.81E−07	0.0016	2.93E−07
	Map 3	1.88E−04	4.81E−07	8.17E−06	4.02E−06
	Map 4	1.89E−04	0.0005	0.0007	8.50E−05
	Map 5	2.69E−09	7.55E−07	3.60E−09	6.25E−08

In simple environments (Maps 1 and 2), as shown in Table 7, the *p*-values demonstrate statistically significant differences in favor of AMET when compared to CME-MGWO, CME-MODA, and CME-MACO. The results against CME-SSA show no significant difference in Map 1 (p = 0.076), suggesting comparable performance, but a significant difference in Map 2. In complex environments (Maps 1 to 5), AMET consistently shows statistically significant advantages over the other algorithms. The particularly low *p*-values (in the order of E-06 to E-09) across most comparisons underline the robustness of AMET in these settings. This indicates that AMET’s superior performance in exploring complex environments is highly unlikely to be due to random variation, reaffirming its effectiveness. These statistical results strongly support the hypothesis that AMET outperforms the other algorithms in terms of exploration efficiency in both simple and complex environments. The significant *p*-values also reinforce the reliability of the conclusions drawn from the visual and numerical analysis previously conducted.

Table 8 presents the *p*-values resulting from the Wilcoxon rank sum test comparing the time efficiency of AMET against other algorithms. Across both simple and complex maps, the *p*-values are consistently very low (in the order of E-11), indicating that the differences in time efficiency between AMET and the comparative algorithms are statistically significant. In simple environments (Maps 1 and 2), as shown in Table 8, AMET demonstrates a significant advantage over CME-MGWO, CME-SSA, CME-MODA, and CME-MACO in terms of time efficiency. These *p*-values strongly suggest that AMET’s faster completion times are not due to random chance but are a result of its efficient algorithmic design. The complex environment results (Maps 1 to 5) mirror these findings, with AMET again showing a significant time efficiency advantage over the other algorithms. The *p*-values highlight the effectiveness of AMET in managing time constraints in more challenging scenarios, where navigating and exploring efficiently is crucial.

**Table 8:** *p*-values from Wilcoxon rank sum test on time efficiency.

Map type	Map No	AMET versus CME-MGWO	AMET versus CME-SSA	AMET versus CME-MODA	AMET versus CME-MACO
Simple	Map 1	3.43E−11	4.36E−11	3.02E−10	2.92E−11
	Map 2	3.74E−11	3.06E−11	3.12E−10	4.13E−11
Complex	Map 1	4.14E−11	3.23E−11	3.42E−11	3.27E−11
	Map 2	3.96E−11	3.32E−11	1.26E−10	2.93E−11
	Map 3	3.47E−11	3.06E−11	2.58E−10	4.53E−10
	Map 4	2.86E−11	4.01E−11	3.01E−11	5.21E−11
	Map 5	4.41E−11	4.06E−11	3.16E−11	3.63E−10

The statistical validation provided by the Wilcoxon rank-sum test reinforces the observed performance trends across different environments. The results confirm that AMET’s superior exploration efficiency and time management are not due to random variation but are statistically significant when compared to alternative algorithms. Particularly in complex environments, the exceptionally low *p*-values emphasize the reliability of AMET’s optimization strategies.

Given these findings, a broader analysis is warranted to contextualize AMET’s advantages in real-world applications. The following section synthesizes the numerical and statistical insights, offering a comprehensive evaluation of AMET’s strengths, limitations, and implications for multi-robot exploration tasks.

### Summary and analysis of results

Tables 1, 2, 3, 4, 5 and 6 present a summary of how each algorithm performed in terms of coverage, time usage, and the chance of failing in simple and complex environments. The proposed AMET algorithm achieved the best coverage scores on all tested maps, which is especially important in environments with many obstacles. It also finished the tests in less time than the other methods, suggesting it can handle time constraints better. Another notable advantage is its small number of failed attempts, meaning AMET ran more reliably, an essential factor for practical applications. To further study these differences, Tables 7 and 8 show the statistical results using the Wilcoxon rank sum test. According to these tests, AMET’s performance in coverage and speed is significantly better than the other algorithms, as indicated by very low *p*-values. This means the improvements offered by AMET, in both area coverage and time efficiency, are not random. Hence, these findings strengthen the idea that the AMET algorithm can be a good solution for robust and efficient multi-robot exploration.

### Practical implications and limitations of AMET

The dual-objective structure of AMET, which jointly optimizes exploration efficiency and mapping accuracy, presents significant advantages for real-world deployment. In search-and-rescue operations or planetary surface exploration, maximizing area coverage is essential for locating survivors or gathering environmental data, while mapping accuracy ensures the information is reliable for navigation, planning, and decision-making. The ability to balance these competing goals makes AMET particularly well-suited to missions where both speed and precision are critical.

However, AMET’s multi-objective nature introduces trade-offs. In highly time-constrained scenarios, such as when real-time decisions must be made with minimal computational overhead, a well-tuned single-objective algorithm may be more appropriate due to faster convergence and lower complexity. Also, AMET may require more careful parameter tuning to maintain optimal balance between objectives, which could be challenging in dynamically changing or resource-constrained environments without prior calibration.

## Conclusion

This study proposed and evaluated AMET by comparing its performance against several well-established multi-robot exploration algorithms, including CME-MGWO, CME-SSA, CME-MODA, and CME-MACO. The comparative analysis was conducted across various environmental complexities, utilizing both qualitative and quantitative assessments. To ensure statistical rigor, the Wilcoxon rank-sum test was applied to evaluate the significance of the observed results.

Experimental findings consistently demonstrated that AMET outperforms the competing algorithms, achieving the highest exploration coverage, shortest task completion times, and lowest failure rates across the majority of test cases. The results indicate that AMET provides superior spatial coverage, improved time efficiency, and enhanced reliability, making it a robust solution for both simple and complex environments. The statistical validation further reinforced these conclusions, as the Wilcoxon rank-sum test produced low *p*-values, confirming that AMET’s superior performance is not due to random variations but rather to its efficient optimization framework. This success is attributed to its multi-objective optimization strategy, which dynamically balances exploration efficiency and mapping accuracy. By integrating adaptive decision-making with coordinated path planning, AMET enables robots to navigate and map unknown environments more effectively than conventional approaches.

In conclusion, AMET has proven to be a highly reliable and efficient framework for autonomous multi-robot exploration. Its ability to adapt to diverse exploration challenges underscores its potential for real-world applications, including search-and-rescue missions, planetary exploration, and large-scale environmental monitoring. Future research should focus on further refining AMET’s optimization mechanisms, expanding its applicability to real-world robotic systems, and incorporating additional performance metrics such as energy efficiency, communication overhead, and scalability. These findings provide a strong foundation for the continued advancement of autonomous exploration systems, contributing to the development of more intelligent and efficient multi-robot platforms.

While AMET has demonstrated strong performance in multi-robot exploration, several areas remain open for further development. One key direction is enhancing adaptability through reinforcement learning, which would allow AMET to adjust its exploration strategy dynamically based on real-time environmental feedback. This would enable the algorithm to refine decision-making over multiple deployments, improving efficiency in unpredictable environments. Another critical aspect is scalability, particularly in large-scale environments with a higher number of robots and more complex navigation constraints. Future work should focus on optimizing inter-robot communication and decentralized coordination, ensuring that AMET can maintain high performance as the problem size increases.

In addition, energy-efficient path planning should be integrated, considering battery consumption and recharge scheduling, which is essential for long-duration exploration missions. Hardware validation in real-world robotic platforms is another crucial step to assess AMET’s robustness under real-time constraints, sensor noise, and imperfect localization. Expanding the multi-objective framework to incorporate collision avoidance, adaptive risk assessment, and terrain adaptability would further improve AMET’s practicality for autonomous exploration in challenging and dynamic environments as well.

Another interesting area worth exploring is the integration of reinforcement learning into the AMET framework to enable adaptive decision-making in highly dynamic environments. Specifically, policy-based reinforcement learning methods could be used to train each robot to dynamically adjust its objective weighting based on environmental context— (for instance, prioritizing mapping accuracy over exploration speed in cluttered regions). This integration would allow AMET to self-tune its trade-off strategy in real time, improving resilience in unpredictable scenarios.

To address scalability, future extensions of AMET could focus on hierarchical coordination mechanisms suitable for large-scale deployments involving hundreds of robots. Future work should also focus on optimizing inter-robot communication and decentralized coordination, which ensures that AMET can maintain high performance as the problem size increases. One promising approach is to group robots into clusters, each led by a coordinator agent running a localized version of AMET. These cluster-level decisions would then be synchronized via a lightweight consensus protocol to ensure global coverage while minimizing inter-robot conflict and communication overhead.

Last but not least, validating AMET in real-world robotic systems could be done as well. We suggest using hardware-in-the-loop testing with mobile robots in indoor environments that simulate real-world complexity. These tests will evaluate AMET’s robustness to sensor noise, partial observability, localization errors, and communication delays. This practical evaluation will provide insight into how AMET can be adapted for deployment in safety-critical domains such as disaster response and autonomous planetary exploration.

## Data Availability

All data generated or analyzed during this study are included in this published article.
